# Choosing sides: unusual ultrafast charge transfer pathways in an asymmetric electron-accepting cyclophane that binds an electron donor†
†Electronic supplementary information (ESI) available: Synthesis, NMR, X-ray crystallography, optical and electrochemical experiments. CCDC 1872160 and 1872161. For ESI and crystallographic data in CIF or other electronic format see DOI: 10.1039/c8sc05514a


**DOI:** 10.1039/c8sc05514a

**Published:** 2019-03-11

**Authors:** Jiawang Zhou, Yilei Wu, Indranil Roy, Avik Samanta, J. Fraser Stoddart, Ryan M. Young, Michael R. Wasielewski

**Affiliations:** a Department of Chemistry , Northwestern University , 2145 Sheridan Road , Evanston , Illinois 60208-3113 , USA . Email: m-wasielewski@northwestern.edu ; Email: ryan.young@northwestern.edu; b Institute for Sustainability and Energy at Northwestern , Northwestern University , 2145 Sheridan Road , Evanston , Illinois 60208-3113 , USA; c Institute for Molecular Design and Synthesis , Tianjin University , Tianjin 300072 , China; d School of Chemistry , University of New South Wales , Sydney , New South Wales 2052 , Australia

## Abstract

Constructing functional molecular systems for solar energy conversion and quantum information science requires a fundamental understanding of electron transfer in donor–bridge–acceptor (D–B–A) systems as well as competitive reaction pathways in acceptor–donor–acceptor (A–D–A) and acceptor–donor–acceptor′ (A–D–A′) systems. Herein we present a supramolecular complex comprising a tetracationic cyclophane having both phenyl-extended viologen (ExV^2+^) and dipyridylthiazolothiazole (TTz^2+^) electron acceptors doubly-linked by means of two *p*-xylylene linkers (TTzExVBox^4+^), which readily incorporates a perylene (Per) guest in its cavity (Per ⊂ TTzExVBox^4+^) to establish an A–D–A′ system, in which the ExV^2+^ and TTz^2+^ units serve as competing electron acceptors with different reduction potentials. Photoexcitation of the Per guest yields both TTz^+^˙–Per^+^˙–ExV^2+^ and TTz^2+^–Per^+^˙–ExV^+^˙ in <1 ps, while back electron transfer in TTz^2+^–Per^+^˙–ExV^+^˙ proceeds *via* the unusual sequence TTz^2+^–Per^+^˙–ExV^+^˙ → TTz^+^˙–Per^+^˙–ExV^2+^ → TTz^2+^–Per–ExV^2+^. In addition, selective chemical reduction of TTz^2+^ gives Per ⊂ TTzExVBox^3+^˙, turning the complex into a D–B–A system in which photoexcitation of TTz^+^˙ results in the reaction sequence ^2^*TTz^+^˙–Per–ExV^2+^ → TTz^2+^–Per–ExV^+^˙ → TTz^+^˙–Per–ExV^2+^. Both reactions TTz^2+^–Per^+^˙–ExV^+^˙ → TTz^+^˙–Per^+^˙–ExV^2+^ and TTz^2+^–Per–ExV^+^˙ → TTz^+^˙–Per–ExV^2+^ occur with a (16 ± 1 ps)^–1^ rate constant irrespective of whether the bridge molecule is Per^+^˙ or Per. These results are explained using the superexchange mechanism in which the ionic states of the perylene guest serve as virtual states in each case and demonstrate a novel supramolecular platform for studying the effects of bridge energetics within D–B–A systems.

## Abstract 2

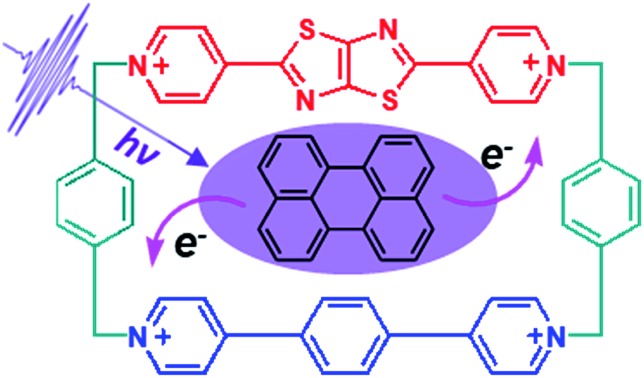
Photo-driven electron transfer is faster from an electron donor guest to the harder to reduce acceptor in an asymmetric cyclophane host.

## Introduction

The study of photoinduced electron transfer reactions in well-defined molecular multi-acceptor systems provides valuable information regarding their use in solar energy conversion.^1^–^14^ Cyclophanes that use two *p*-xylyl groups to connect two electron-deficient units to form a box-like structure are well known to strongly bind electron-rich hydrocarbons inside their cavities,^15^,^16^ and thus these supramolecular complexes are important for developing an understanding of photoinduced electron transfer reactions in acceptor–donor–acceptor (A–D–A) systems. Previous work has demonstrated efficient electron transfer in host–guest complexes based on symmetrical electron-deficient phenyl-extended viologen^17^ (ExV^2+^)- and perylenediimide^18^ (PDI)-based cyclophanes, in which a perylene (Per) guest molecule serves as the electron donor. In contrast, competitive electron transfer within asymmetric A–D–A′ π–π stacks is more complicated, and presents both synthetic and spectroscopic challenges. However, understanding the underlying factors that govern competitive electron transfer reactions in A–D–A′ systems is important for developing multi-pathway electron transfer systems^19^–^23^ for quantum information science as well as solar energy harvesting and storage. Herein, we show that an asymmetric cyclophane incorporating two electron acceptor subunits with different reduction potentials is capable of hosting a Per electron donor in its cavity, and thus can serve as an excellent molecular platform for this purpose.

Our earlier work on oligomeric π-conjugated bridges, such as *p*-phenylenevinylene,^24^*p*-phenylene^25^ and fluorene^26^ has revealed the importance of the bridge states in determining the electron transfer rate *via* the superexchange mechanism.^27^ While providing valuable information, these covalently linked D–B–A systems usually demand laborious multistep syntheses, so that asymmetric cyclophane host acceptors with easily exchangeable guest donors bound to the host by supramolecular forces are appealing alternatives. An early approach using a variation of this concept employed C-shaped donor–acceptor molecules to trap solvent molecules or to hang pendant bridge molecules between the donor and the acceptor for studying superexchange involving the solvent and/or the bridge.^28^–^32^ In addition, hemicarcerands containing a variety of guest molecules have been used to study electron transfer between the guest and semiconductor nanoparticles^33^ or zinc porphyrin-substituted cytochromes.^34^

**Fig. 1:**
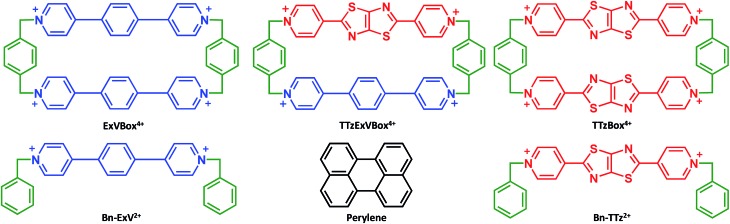
Structural formulas of compounds used in this investigation.

Recently we reported the synthesis and application in cell imaging of a hybrid cyclophane, TTzExVBox^4+^ (Fig. 1), which contains an ExV^2+^ and a dipyridylthiazolothiazole (TTz^2+^) unit.^35^ In the study reported here, Per was chosen as the electron donor because its photophysical properties have been thoroughly investigated,^36^,^37^ and it can be readily encapsulated by the cyclophane to form the Per ⊂ TTzExVBox^4+^ complex (*vide infra*). Importantly, the Per first excited singlet state (^1^*Per) has been previously shown to exhibit fast electron transfer to ExV^2+^ within the corresponding symmetric cyclophane ExVBox^4+^ (also called ExBox^4+^ in earlier publications).^17^ Since the reduction potential of TTz^2+^ is 0.4 eV more positive than ExV^2+^ (*vide infra*), photoreduction of TTz^2+^ by ^1^*Per is expected to be thermodynamically accessible as well. Therefore, we expect the asymmetric Per ⊂ TTzExVBox^4+^ complex to be suitable as an A–D–A′ model system to study competitive two-pathway photoinduced reactions.

**Scheme 1:**
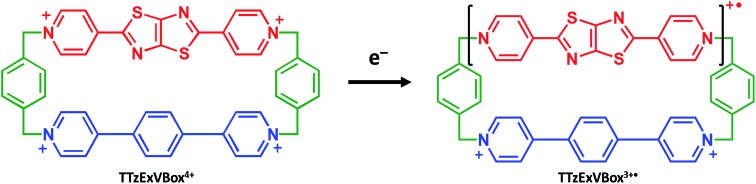
Chemical reduction of TTzExVBox^4+^ to TTzExVBox^3+^˙.

Furthermore, we show that the A–D–A′ system of Per ⊂ TTzExVBox^4+^ can be easily transformed into a D–B–A system by selective chemical reduction of TTz^2+^ to TTz^+^˙ wherein the lowest excited doublet state of TTz^+^˙ (^2^*TTz^+^˙) serves as the donor within the cyclophane (Scheme 1). Photoexcited radical anions of polycyclic aromatic molecules can act as strong reductants;^38^–^42^ we show that this is also true for the TTz^+^˙ radical cation, which makes the Per ⊂ TTzExVBox^3+^˙ complex a useful D–B–A system for studying the role of a non-covalently linked bridge unit in electron transfer reactions initiated from excited doublet states.

Here we investigate competitive intramolecular charge transfer (ICT) dynamics in two complexes – namely, Per ⊂ TTzExVBox^4+^ (A–D–A′) and Per ⊂ TTzExVBox^3+^˙ (D–B–A) - in CH_3_CN by means of femtosecond transient visible and NIR absorption (fsTA) spectroscopy. Photoexcitation of the Per guest in Per ⊂ TTzExVBox^4+^ results in competitive electron transfer to both ExV^2+^ and TTz^2+^ subunits. We observe that the ExV^+^˙ population is approximately twice that of TTz^+^˙, despite the fact that the free energy of reaction for the TTz^+^˙ formation is 0.4 eV more negative than that for ExV^+^˙. In control experiments, the forward adiabatic electron transfer (FET) rate for Per^+^˙–ExV^+^˙ ion-pair formation is found to be about two times faster than that for TTz^+^˙–Per^+^˙, indicating that although the barriers for both reactions are low, the latter electron transfer reaction likely occurs through a slightly higher barrier. The subsequent back electron transfer (BET) pathway for Per^+^˙–ExV^+^˙ is very unusual. The BET sequence is TTz^2+^–Per^+^˙–ExV^+^˙ → TTz^+^˙–Per^+^˙–ExV^2+^ → TTz^2+^–Per–ExV^2+^, where electron transfer occurs initially from ExV^+^˙ to TTz^2+^, bypassing conventional direct BET to Per^+^˙, so that the electron transfer rate for this first step through the Per^+^˙ bridge is (16 ± 1 ps)^–1^. In comparison, following selective photoexcitation of TTz^+^˙ in Per ⊂ TTzExVBox^3+^˙ the electron transfer sequence is ^2^*TTz^+^˙–Per–ExV^2+^ → TTz^2+^–Per–ExV^+^˙ → TTz^+^˙–Per–ExV^2+^. The electron transfer rate of the second step through the Per bridge is also (16 ± 1 ps)^–1^, so that irrespective whether the bridge molecule is Per^+^˙ or Per, the electron transfer rates through the bridge are the same. The strong influence of the Per bridge on the electron transfer rates is further demonstrated by a control experiment with the TTzExVBox^3+^˙ cyclophane itself without the Per guest, where the rate of the TTz^2+^–ExV^+^˙ → TTz^+^˙–ExV^2+^ BET reaction is about 8 times slower than that of the TTz^2+^–Per–ExV^+^˙ → TTz^+^˙–Per–ExV^2+^ reaction measured in Per ⊂ TTzExVBox^3+^˙. Despite their unusual nature, these results can be explained in the context of the superexchange mechanism^27^ (*vide infra*), where the electron transfer rates in these systems are controlled by mixing the donor and acceptor states with the closely lying virtual states of the guest molecule. This work shows how A–D–A′ and D–B–A π–π stacked systems can be conveniently realized by using supramolecular host–guest complexes to explore electron transfer mechanisms.

## Experimental section

### Synthesis

The synthesis and characterization of the compounds studied here are described in the ESI.†

### Steady-state optical spectroscopy

UV-Vis-NIR absorption spectra were acquired on a Shimadzu UV-3600 spectrophotometer. UV-Vis titrations were performed by adding small volumes of a concentrated cyclophane solution in CH_3_CN to a solution of Per in CH_3_CN. The appearance of the lower energy CT band was used to determine the association constant (*K*_a_). Assuming a 1 : 1 complexation mode, *K*_a_ = 1.0 ± 0.2 × 10^5^ M^–1^ was calculated using Dynafit,^43^ a program which employs nonlinear least-squares regression on receptor-substrate binding data measured by UV-Vis spectroscopy titration experiments. Absolute photoluminescence quantum yields were determined using a HORIBA Nanolog spectrofluorimeter equipped with an integrating sphere. All samples were dissolved in CH_3_CN unless noted otherwise. Chemically reduced samples were prepared using cobaltocene (CoCp_2_) as the reducing agent under a N_2_ atmosphere.

### Transient absorption spectroscopy

The fsTA spectroscopy apparatus has been described previously,^17^ and here we present details specific to the present work. The 620 nm photoexcitation pulses were obtained using a home-built optical parametric amplifier pumped by 414 nm pump pulses generated by frequency-doubling the 828 nm fundamental in a lithium triborate (LBO, *θ* = 90°, *φ* = 31.7°, 1 mm) crystal.^44^ The energy of the photoexcitation pulses was attenuated to ∼1 μJ per pulse using neutral density filters and focused to a 200 μm spot size at the sample. The pump polarization was randomized using a commercial depolarizer (DPU-25-A, Thorlabs, Inc.) to eliminate any orientational dynamics contributions from the experiment. FsTA spectra were collected on a commercial spectrometer (customized Helios, Ultrafast Systems LLC). The path length of the quartz cuvettes was 2 mm, and the sample concentration of the cyclophanes was approximately 3 × 10^–4^ M to yield a typical optical density at the excitation wavelength of about 0.5. All samples were stirred to avoid localized heating or degradation effects during optical measurements.

## Results and discussion

### Supramolecular complex formation

**Fig. 2:**
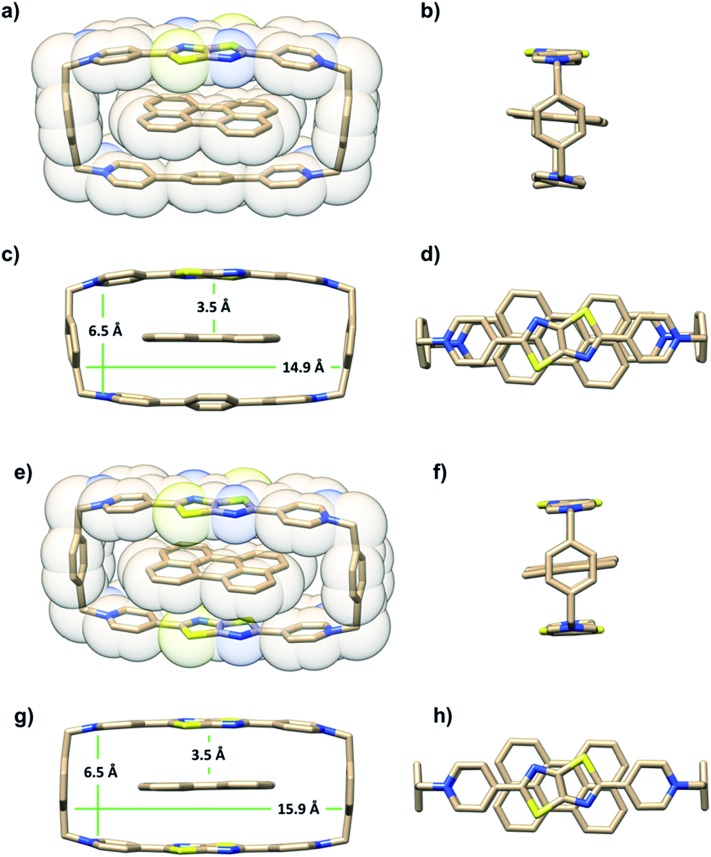
A blend of tubular and space-filling representations of the solid-state structure of (a–d) Per ⊂ TTzExVBox^4+^ and (e–h) Per ⊂ TTzBox^4+^ showing the main structural parameters. Counterions and residual solvent molecules are omitted for clarity.

The ability of either TTzExVBox^4+^ or TTzBox^4+^ to form inclusion complexes with a Per guest is first demonstrated by the XRD analysis of single crystals (see ESI†). Fig. 2 shows the superstructure of host–guest complexes of the two cyclophanes with Per viewed from different angles. The dimensions of the cavity of TTzExVBox^4+^ and TTzBox^4+^ are approximately 7 Å wide × 15 Å long. These values are similar to those of reported for ExVBox^4+^, which is known to strongly bind polycyclic aromatic hydrocarbons as a result of significant van der Waals interactions at the inherent 3.5 Å π–π stacking distances between the electron acceptor units of the tetracationic cyclophane and the electron donor guests.^15^ The formation of the Per ⊂ TTzExVBox^4+^ and Per ⊂ TTzBox^4+^ complexes in solution is further corroborated by their ^1^H NMR spectra (Fig. S3†), showing significant upfield shifts of the aromatic proton resonances of TTz^2+^ and ExV^2+^ as well as a downfield shift of the *p*-xylylene proton resonances of the cyclophanes. These chemical shift changes can be explained by π-electron shielding of the face-to-face oriented aromatic rings upon complexation and provides evidence for Per ⊂ TTzExVBox^4+^ formation in solution. Importantly, the Per peaks are significantly broadened, which suggests they sample a distribution of magnetic environments in solution.

### Steady-state electronic spectra

**Fig. 3:**
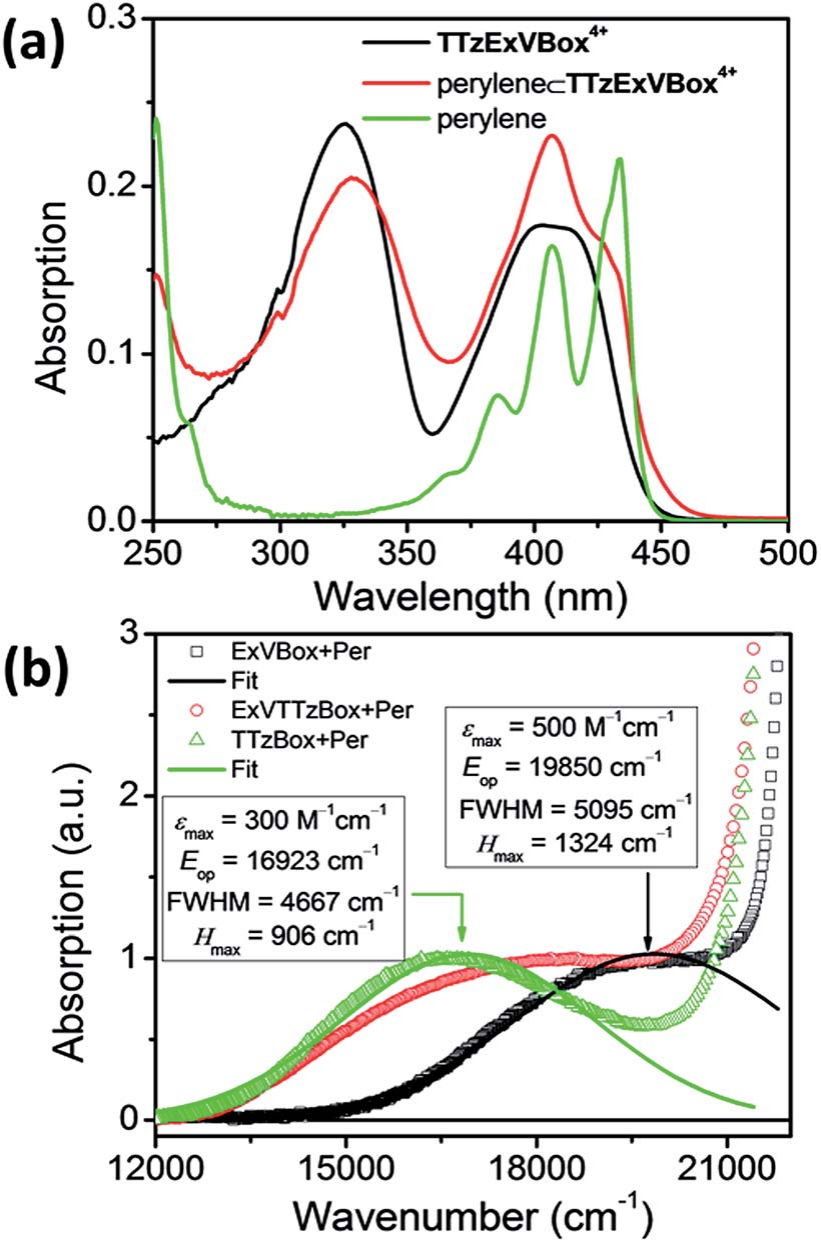
(a) Steady-state absorption spectra of TTzExVBox^4+^, Per and Per ⊂ TTzExVBox^4+^ in CH_3_CN. The concentration of Per in the latter two solutions is kept the same. (b) CT bands in Per ⊂ ExVBox^4+^, Per ⊂ TTzBox^4+^, and Per ⊂ TTzExVBox^4+^, and extracted couplings.

The steady-state absorption spectrum of TTzExVBox^4+^, shown in Fig. 3a and S4,† is comprised of two distinct absorption bands centered around 330 and 405 nm, associated with the localized π–π* transitions of the ExV^2+^ and TTz^2+^ subunits,^45^ respectively. The fluorescence quantum yield of TTzExVBox^4+^ approaches unity (*Φ*_f_ = 0.94 ± 0.02), indicating that no competitive quenching due to FET occurs within the cyclophane.

The Per ⊂ TTzExVBox^4+^ samples were prepared by adding a TTzExVBox^4+^ solution in CH_3_CN into a saturated Per solution in CH_3_CN. The formation of the Per ⊂ TTzExVBox^4+^ complex is evidenced by the appearance of a weak CT band centered around 560 nm, indicative of an electronic interaction between the host and guest (Fig. 3b). Similar CT bands are observed in complexes of perylene and the symmetric ExVBox^4+^ and TTzBox^4+^ cyclophanes, also shown in Fig. 3b. The electronic coupling between the ground states and the charge-separated states in Per ⊂ ExVBox^4+^ and Per ⊂ TTzBox^4+^ can be extracted from these spectra using Mulliken–Hush theory^46^,^47^ as 1324 and 906 cm^–1^, respectively. Such strong coupling indicates that ICT is likely adiabatic. Additionally, the CT band in Per ⊂ TTzExVBox^4+^ appears as a superposition of bands of the two symmetric complexes, which suggests that those complexes share similar electronic coupling to that of the asymmetric complex and that they can serve as suitable controls for understanding its photophysics.

It is important to point out that the solution-phase optical experiments are ensemble measurements, and since the host–guest binding is dynamic, they sample a distribution of Per–cyclophane orientations. The broadened Per peaks in the NMR spectra indicate fast exchange on the timescale of that experiment, such that the optical experiments sample different geometries at different stages of the exchange. These geometries are distributed about the minimum energy structure shown in Fig. 2.

Quantitative relative extinction coefficients of ExV^+^˙ and TTz^+^˙ were obtained by adding equimolar amounts of CoCp_2_ to the monomeric reference compounds, Bn-ExV^2+^ and Bn-TTz^2+^, respectively (Fig. S5†). Bn-ExV^+^˙ shows major absorption bands at 474, 513, 965 and 1110 nm, as reported previously,^17^,^48^,^49^ while Bn-TTz^+^˙ shares a similar absorption pattern with corresponding red shifts of the absorption bands to 557, 612, 1105 and 1305 nm. Since the absorption peaks at 965 nm for Bn-ExV^+^˙ and 1305 nm for Bn-TTz^+^˙ do not overlap significantly with each other or with the Per S_*n*_ ← S_1_ absorption, their relative extinction coefficients at those wavelengths were used to estimate their relative reduction yields *via* photoinduced electron transfer (*vide infra*).

Per ⊂ TTzExVBox^3+^˙ was prepared by addition of a sub-stoichiometric amount of CoCp_2_ (Fig. S6†). The TTz^+^˙ absorption bands at 629, 1130 and 1344 nm in Per ⊂ TTzExVBox^3+^˙ are further red-shifted compared to those of TTzExVBox^3+^˙ at 620, 1112 and 1315 nm, again indicating an electronic interaction between the guest and partially reduced host.

### Forward electron transfer in Per ⊂ TTzExVBox^4+^

Based on the relatively mild reduction potentials of the ExV^2+^ and TTz^2+^ subunits, it is reasonable to expect competitive charge transfer from ^1^*Per to each acceptor subunit. The first reduction potentials of ExV^2+^ and TTz^2+^ in TTzExVBox^4+^ are *E*_red_ = –0.75 and –0.35 V *vs.* Ag/AgCl, respectively, in CH_3_CN, which shows that the reduction of the TTz^2+^ unit is favored.^35^ The corresponding Per oxidation potential is *E*_ox_ = 1.01 V *vs.* Ag/AgCl and the ^1^*Per energy is *E*_S_ = 2.85 eV.^17^ Given that the experiments were performed in CH_3_CN, which has a high dielectric constant (*ε* = 38), the free energies for charge separation from ^1^*Per to the ExV^2+^ and TTz^2+^ subunits are estimated as Δ*G*_FET_ ≅ *E*_ox_ – *E*_red_ – *E*_S_ = –1.09 and –1.49 eV, respectively.^50^ The electronic couplings between ^1^*Per and each acceptor are expected to be large as well, owing to the large π-overlap between the donor guest and acceptor host.

**Fig. 4:**
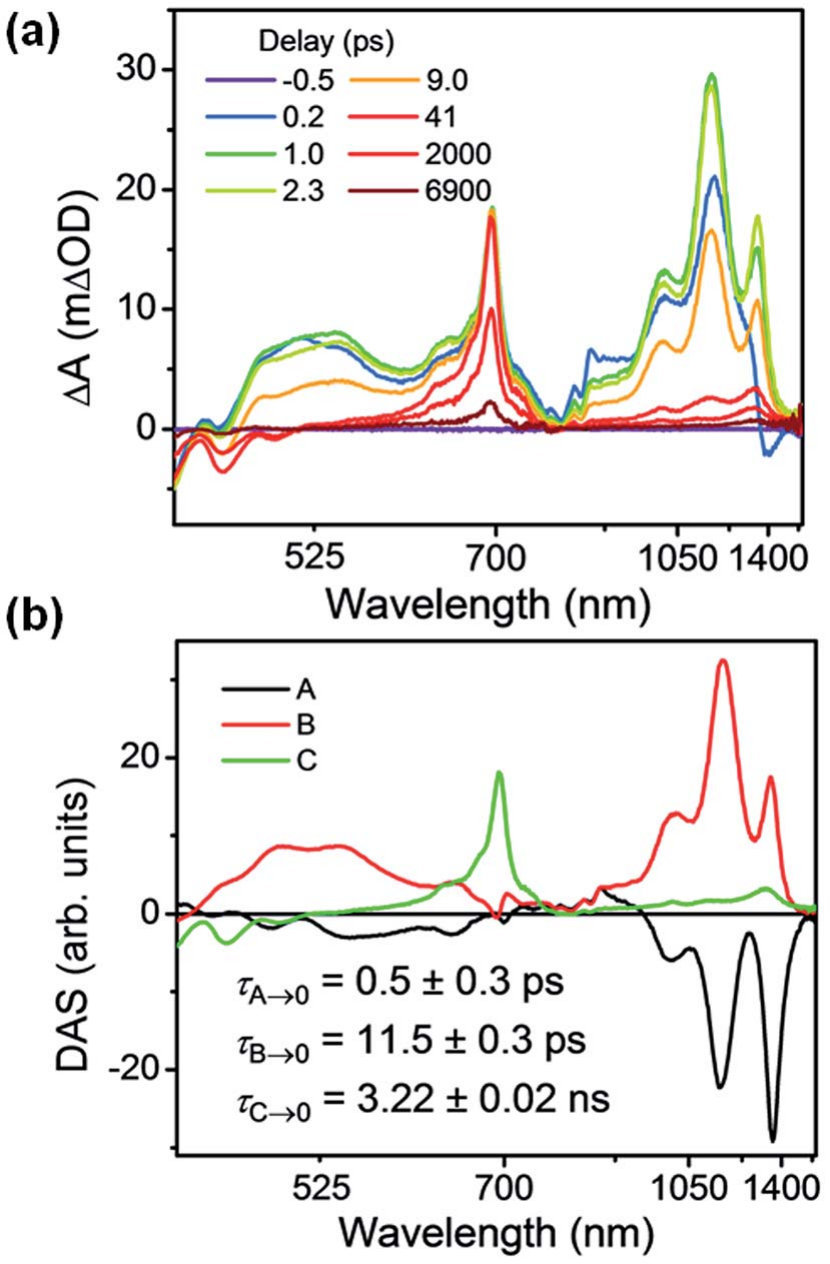
(a) fsTA spectra and (b) decay-associated spectra (DAS) of Per ⊂ TTzExVBox^4+^ in CH_3_CN excited at 414 nm ((A) formation of Per^+^˙–TTz^+^˙, (B) decay of Per^+^˙–TTz^+^˙, (C) decay of unbound ^1^*Per). The wavelength axis is plotted in reciprocal space. Time constants are for the representative data set shown here; averages and standard deviations from multiple experiments are given in Table 1.

FsTA spectroscopy was used to probe the charge transfer dynamics of Per ⊂ TTzExVBox^4+^ upon photoexcitation of the Per guest at 414 nm (Fig. 4a). Rapid electron transfer from ^1^*Per to both ExV^2+^ and TTz^2+^ subunits is observed within 1 ps, as indicated by the appearance of the characteristic absorption bands for ExV^+^˙ (1010 and 1160 nm) and TTz^+^˙ (1160 and 1340 nm). The broad and less-structured absorption from 500 to 700 nm can be ascribed to the overlapping absorption features of ExV^+^˙ and TTz^+^˙ in visible region, as well as Per^+^˙. In the next 50 ps all ExV^+^˙ and TTz^+^˙ bands disappear, and the spectra are dominated by a sharp excited-state absorption (ESA) feature at 700 nm, along with weak bleach and stimulated emission features around 440 nm. These signals persist beyond the FET time and decay within 7 ns, and can be assigned to a population of unbound Per in solution that is excited in parallel with the complex. We do not observe significant co-excitation of the TTz^2+^ unit, likely owing to the higher concentration and extinction coefficient at 414 nm of the excess perylene in solution.

Global analysis was used to deconvolute the fsTA spectra into its component decay-associated spectra (DAS) (Fig. 4b). Details of the fitting methodology are given in the ESI (Fig. S7†). Three components were necessary to adequately fit the data. The first component with a 0.5 ± 0.3 ps lifetime is assigned to the competitive FET process. The second component describes the ensuing BET in *τ*_BET_ = 11.4 ± 0.5 ps, while the third component captures the ^1^*Per decay of unbound Per in *τ*_S_1__ = 3.6 ± 0.3 ns, consistent with our previous measurements,^17^ and with the 3.9 ns timescale obtained from Per in CH_3_CN (Fig. S13†).

Given that the free energy of reaction for electron transfer from ^1^*Per to TTz^2+^ is 0.4 eV more negative than to ExV^2+^, we might expect to see a larger TTz^+^˙ population with respect to ExV^+^˙. However, the fsTA spectra at early times show that the population of ExV^+^˙ relative to that of TTz^+^˙ is about 2 : 1, based on an independent measurement of the intensities of the 1010 nm band for ExV^+^˙ and the 1340 nm band for TTz^+^˙ and their relative extinction coefficients discussed above. If the relative coupling strengths observed in the CT spectra are preserved in the perylene excited state, then the FET process should be adiabatic. Indeed, the FET rates in each of the symmetric host–guest complexes are all very fast: in Per ⊂ ExVBox^4+^ FET occurs in *τ*_FET_ < 0.3 ps,^17^ whereas in Per ⊂ TTzBox^4+^*τ*_FET_ < 0.5 ps (Fig. S8†). This implies that the barrier for FET to TTz^2+^ is slightly larger than that to ExV^2+^, which itself may be barrierless.^17^ The actual intrinsic FET rate in the presence of one electron acceptor should be two times slower than the statistical rate observed with two equivalent acceptors. In principle, the FET rate in Per ⊂ TTzExVBox^4+^ can be estimated by calculating the sum of the intrinsic rate constants observed for Per ⊂ ExVBox^4+^ and Per ⊂ TTzBox^4+^ systems, which is (0.4 ps)^–1^ in this case and is close to the observed (0.5 ps)^–1^ in the Per ⊂ TTzExVBox^4+^ complex. We note that the time resolution of the fsTA experiments is about 0.3 ps, a value that contributes the indicated uncertainty to the determination of these ultrafast FET rates. Nevertheless, the rate difference in the control complexes supports competitive charge transfer in Per ⊂ TTzExVBox^4+^ favoring formation of ExV^+^˙ over TTz^+^˙. The FET rate in Per ⊂ ExVBox^4+^ is at least two times faster than in Per ⊂ TTzBox^4+^, which is in good agreement with the 2 : 1 population ratio in Per ⊂ TTzExVBox^4+^ using relative extinction coefficient analysis. While the first excited state of the TTz radical, ^2^*TTz^+^˙, is about –0.59 eV lower than ^1^*Per (Fig. S5†) and thus an energetically accessible pathway for FET, we do not observe any buildup of such a state. If such an intermediate is populated then FET must occur to it with the observed ∼0.8 ps time constant and be subsequently followed by much more rapid internal conversion down to the lowest state of TTz^+^˙, resulting in the same observed 2 : 1 ratio of ExV^+^˙ : TTz^+^˙.

### Back electron transfer in Per ⊂ TTzExVBox^4+^

**Fig. 5:**
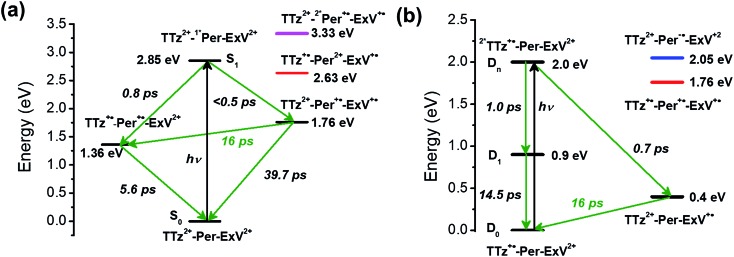
Energy level diagrams of (a) Per ⊂ TTzExVBox^4+^ and (b) Per ⊂ TTzExVBox^3+^˙ in CH_3_CN, in which the zero energy level is taken to be the Per S_0_ state in (a) and the TTz^+^˙ D_0_ state in (b), respectively. The ICT pathways from ExV^+^˙ to TTz^2+^ as well as the corresponding timescales are highlighted in red. The states involved in superexchange mixing are shown in red, blue, and magenta.

Given the ultrafast rates of all the observed electron transfer reactions following photoexcitation of Per within Per ⊂ TTzExVBox^4+^, the resulting radical ion pairs are born in their singlet states and have magnetic spin–spin interactions that are sufficiently strong to prevent spin evolution to produce the corresponding triplet radical ion pairs;^51^,^52^ thus, there are no discernible spin restrictions on any of these reactions. Since the two reduced electron acceptors ExV^+^˙ and TTz^+^˙ observed at 1160 and 1340 nm, respectively, decay with the same apparent time constant, *τ*_BET_ = 11.4 ± 0.5 ps, while the BET reactions in the symmetric cyclophanes Per ⊂ ExVBox^4+^ and Per ⊂ TTzBox^4+^ occur in *τ*_BET_ = 39.7 ± 0.3 ps (ref. 17) and *τ*_BET_ = 5.6 ± 0.3 ps (Fig. S8†), respectively, the decay of the higher energy TTz^2+^–Per^+^˙–ExV^+^˙ intermediate must involve a more rapid competitive pathway. We propose that this pathway involves the ICT reaction sequence TTz^2+^–Per^+^˙–ExV^+^˙ → TTz^+^˙–Per^+^˙–ExV^2+^ → TTz^2+^–Per–ExV^2+^, in which the ionic states of Per^+^˙ are now acting as the bridge states in an ICT superexchange mechanism (*vide infra*). The rate of the first ICT step (*k*_ICT1_) can be determined from *k*_ICT1_ = *k*_obs_ – *k*_ExV_, where *k*_obs_ = (11.4 ± 0.5 ps)^–1^ and *k*_ExV_ = (39.7 ± 0.3 ps)^–1^ for Per ⊂ ExVBox^4+^.^17^ Thus, *k*_ICT1_ = (16 ± 1 ps)^–1^ is slower than *k*_TTz_ = (5.6 ± 0.3 ps)^–1^, the intrinsic BET rate for the symmetric cyclophane Per ⊂ TTzBox^4+^, which results in inverted kinetics and is consistent with the apparent simultaneous decay of ExV^+^˙ and TTz^+^˙. Without such a sequential charge-shift reaction, each population would decay with its own intrinsic rate constant, resulting in biexponential charge recombination, which is not observed. The dynamics are summarized in Fig. 5a.

This unusual BET pathway is also consistent with the overall reaction energetics. Even though BET from ExV^+^˙ directly to Per^+^˙ has a larger free energy of reaction (Δ*G*_BET_ = –1.76 eV), and ExV^+^˙ is much closer to Per^+^˙ than to TTz^2+^, the observed rate is much slower. The BET rates in the symmetric cyclophanes Per ⊂ ExVBox^4+^ and Per ⊂ TTzBox^4+^ can be understood within the context of adiabatic electron transfer theory for mixed-valence systems.^53^,^54^ The rate of back electron transfer *k*_BET_ through a barrier is given by eqn (1a), and the barrier height ΔG* is given by (1b):1a
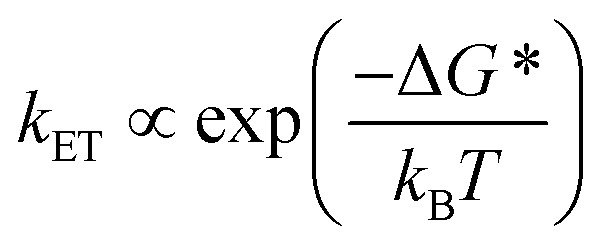

1b
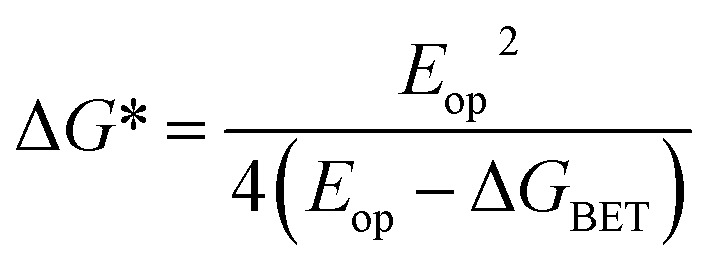
where *E*_op_ is the energy of the optical CT transition (Fig. 3b), and Δ*G*_BET_ is the free energy of back electron transfer (*vide supra*). This analysis shows that ΔG* for BET in Per ⊂ TTzBox^4+^ is 0.32 eV, while that for Per ⊂ ExVBox^4+^ is slightly higher, 0.37 eV, and because of the exponential dependence of the tunneling rate on the barrier height, results in the slower BET time for Per^+^˙ ⊂ ExVBox^3+^˙ (39.7 ps) to the Per^+^˙ ⊂ TTzBox^3+^˙ (5.6 ps).

However, in Per ⊂ TTzExVBox^4+^, the role of the Per^+^˙ guest in the TTz^2+^–Per^+^˙–ExV^+^˙ → TTz^+^˙–Per^+^˙–ExV^2+^ reaction remains unclear. To investigate this process further, we examined the case in which the Per guest acts as the bridge molecule in a donor–bridge–acceptor (D–B–A) configuration.

### Forward and back electron transfer in TTzExVBox^3+^˙

**Fig. 6:**
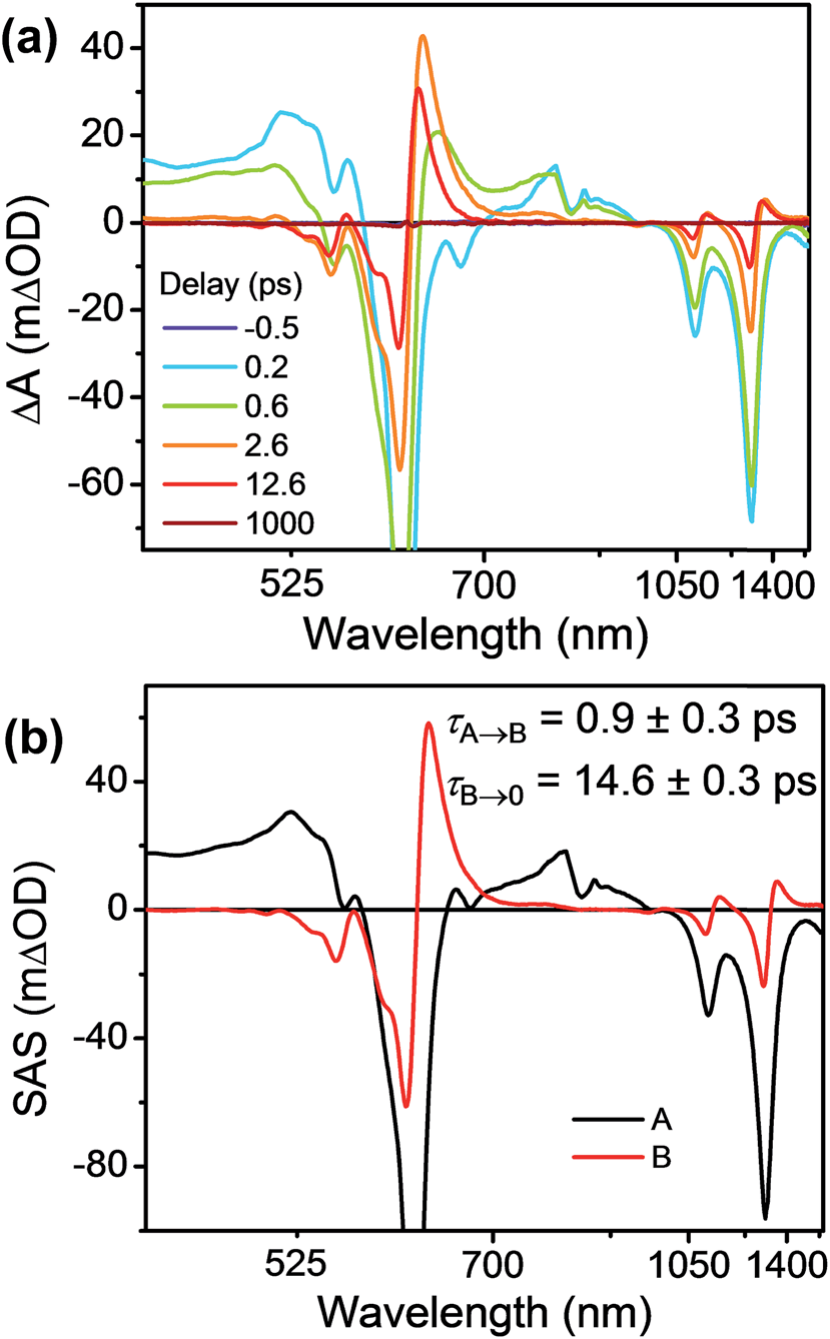
(a) fsTA spectra and (b) species-associated spectra (SAS) of Bn-TTz^+^˙ in CH_3_CN excited at 620 nm ((A) ^2^*TTz^+^˙ (D_*n*_), (B) ^2^*TTz^+^˙ (D_1_)). The wavelength axis is plotted in reciprocal space. Time constants are for the representative data set shown here; averages and standard deviations from multiple experiments are given in Table 1.

FsTA experiments were first performed on the control compound Bn-TTz^+^˙ (Fig. 6a) in order to establish the intrinsic excited-state dynamics of the excited doublet state ^2^*TTz^+^˙. Specifically, Bn-TTz^+^˙ was selectively excited at its 620 nm (2.0 eV) absorption, which is the D_*n*_ ← D_0_ transition of TTz^+^˙. At early times, the spectra consist of two broad ESA features from 400 to 560 nm and from 700 to 950 nm, which overlap with the ground-state bleach (GSB) centered at 610, 1105 and 1305 nm. After 1 ps, the two ESA bands in the visible region merge into a single band around 630 nm, and new ESA bands appear at 1140 and 1360 nm, all of which overlap with the GSB. Both signals decay completely within 100 ps. Global fits to these fsTA data with a species-associated model reveal two time constants, *τ* = 1.0 ± 0.3 and *τ* = 14.5 ± 0.3 ps (Fig. 6b and S9†). Based on the derivative-like lineshape of the NIR ESA band, the fast time constant is assigned to internal conversion down to a vibrationally hot ground electronic state Dhot0, while the slow time constant may be ascribed to vibrational cooling of Dhot0 back to D_0_. However, since Bn-^2^*TTz^+^˙ is not strongly emissive, it is difficult to ascertain the exact internal conversion pathway.

The FET process from ^2^*TTz^2+^ to ExV^+^˙ within TTzExVBox^3+^˙ was examined by first preparing TTz^+^˙ by adding a sub-stoichiometric amount of CoCp_2_ to TTzExVBox^4+^, which results in selective reduction of TTz^2+^ to TTz^+^˙, while ExV^2+^ remains unaffected. The absorption spectrum of Bn-TTz^+^˙ (Fig. S5†) shows that the ^2^*TTz^+^˙ energy is 0.9 eV above TTz^+^˙. In addition, the difference in LUMO energies between TTz^2+^ and ExV^2+^ is 0.4 eV.^35^ Consequently, if the D_1_ or D_*n*_ states of ^2^*TTz^+^˙ in TTzExVBox^3+^˙ are populated, the free energy change for electron transfer from ^2^*TTz^+^˙ to ExV^2+^ is at least –0.5 eV, though this number would be smaller for Dhot0. For the subsequent BET from ExV^+^˙ to TTz^2+^ Δ*G*_BET_ = –0.4 eV, which is directly analogous to the BET process described above for Per ⊂ TTzExVBox^4+^.

**Fig. 7:**
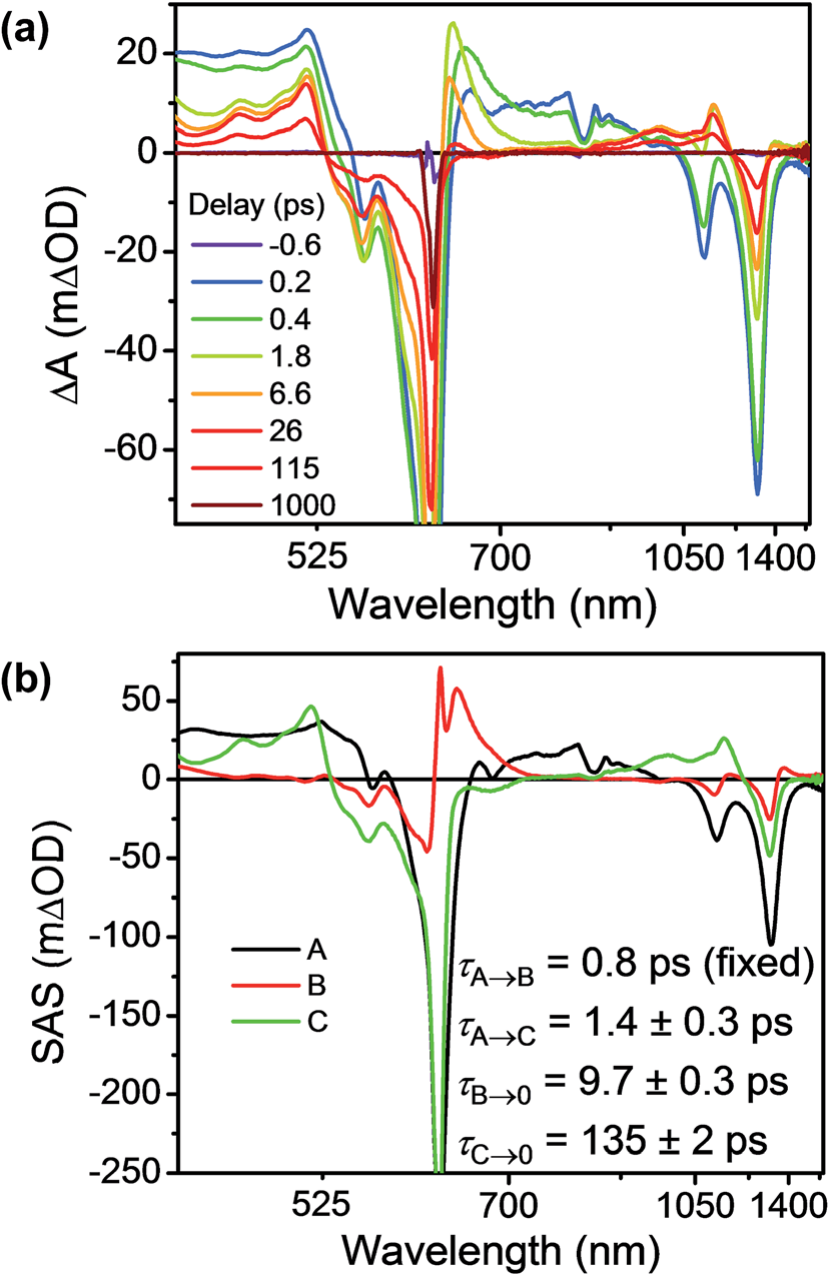
(a) fsTA spectra and (b) species-associated spectra (SAS) of TTzExVBox^3+^˙ in CH_3_CN excited at 620 nm ((A) ^2^*TTz^+^˙ (D_*n*_), (B) ^2^*TTz^+^˙ (D_1_), (C) TTz^2+^-ExV^+^˙). The wavelength axis is plotted in reciprocal space. Time constants are for the representative data set shown here; averages and standard deviations from multiple experiments are given in Table 1.

Having established the ^2^*TTz^+^˙ excited state dynamics, we then investigated the behavior of TTzExVBox^3+^˙ upon selective excitation of TTz^+^˙ at 620 nm (Fig. 7a). Importantly, this wavelength is not resonant with any electronic transitions of other species in TTzExVBox^3+^˙; thus, monitoring the electron transfer sequence ^2^*TTz^+^˙–ExV^2+^ → TTz^2+^–ExV^+^˙ → TTz^+^˙–ExV^2+^ is straightforward. The excited-state dynamics closely resemble those of Bn-TTz^+^˙ at early times with two major GSB features associated with the D_1_ ← D_0_ and D_*n*_ ← D_0_ transitions in the NIR and visible regions, as well as two broad ESA bands. In the following few ps, the GSB intensity decreases and the 630 nm ESA band of the Dhot0 state of Bn-TTz^+^˙ appears. Meanwhile, the characteristic ExV^+^˙ bands appear at 516 and 1145 nm and the TTz^2+^ absorption peak emerges around 405 nm, which unambiguously demonstrates that electron transfer from ^2^*TTz^+^˙ to ExV^2+^ occurs. The simultaneous appearance of the ^2^*TTz^+^˙ (Dhot0) and ExV^+^˙ features suggests that there are two competitive relaxation pathways for ^2^*TTz^+^˙ (D_*n*_), one being internal conversion to the hot ground doublet state and the other being FET to ExV^2+^. These data can be fit with a parallel A → (B, C) → GS species-associated model (Fig. 7b and S10†), which provides three time constants, *τ*_A_ = 1.4 ± 0.3, *τ*_B_ = 8 ± 1 and *τ*_C_ = 135 ± 2 ps. The 8 ps time constant is assigned to Dhot0 → D_0_ cooling by analogy to what is observed for Bn-TTz^+^˙, while the remaining two time constants are assigned to FET and BET, respectively (Fig. 5b). See ESI† for details.

### Forward and back electron transfer in Per ⊂ TTzExVBox^3+^˙

**Fig. 8:**
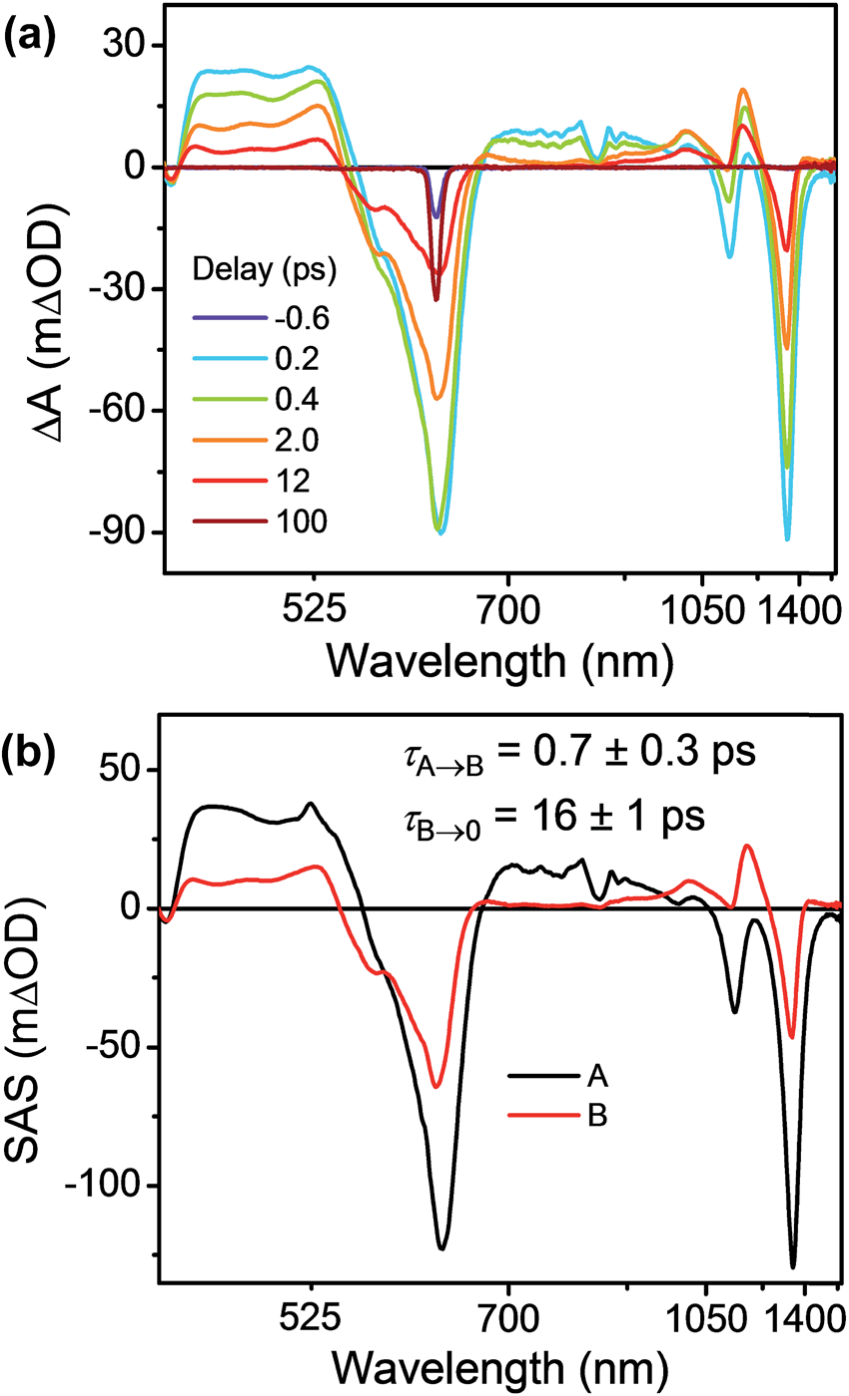
(a) fsTA spectra and (b) species-associated spectra (SAS) of Per ⊂ TTzExVBox^3+^˙ in CH_3_CN excited at 620 nm ((A) ^2^*TTz^+^˙ (D_*n*_), (B) TTz^2+^–Per–ExV^+^˙). The wavelength axis is plotted in reciprocal space. Time constants are for the representative data set shown here; averages and standard deviations from multiple experiments are given in Table 1.

As discussed above there is significant electronic coupling between the host cyclophane and the Per guest in both the ground and excited states, so that the ICT dynamics are likely strongly affected by the presence of the guest. Selective photoexcitation of TTz^+^˙ within Per ⊂ TTzExVBox^3+^˙ with a 620 nm laser pulse results in population of the ^2^*TTz^+^˙ (D_*n*_) state. In contrast to the TTzExVBox^3+^˙ cyclophane host alone, no internal conversion is discernable in the complex; instead, ExV^+^˙ quickly appears in the transient spectra followed by BET back to the ground state. There is no spectral evidence for the formation of the TTz^+^˙–Per^–^˙–ExV^2+^ intermediate. Global fitting of this data set with a sequential A → B → GS species-associated model (Fig. 8b and S11†) gives two time constants, *τ*_A_ = 0.7 ± 0.3 and *τ*_B_ = 16 ± 1 ps, that are assigned to FET and BET, respectively. The presence of Per markedly increases the FET rate, which outcompetes the parallel D_*n*_ → Dhot0 relaxation, and therefore no ^2^*TTz^+^˙ (Dhot0) feature is observed. The *τ*_B_ = 16 ± 1 ps BET time constant in Per ⊂ TTzExVBox^3+^˙ is identical to the 16 ± 1 ps time constant for the analogous charge shift calculated from the measured rate constant for Per ⊂ TTzExVBox^4+^ and its control experiments described above.

As summarized in Table 1, incorporation of Per into TTzExVBox^3+^˙ increases the FET and BET by factors of 2 ± 1 and 8.3 ± 0.5, respectively. This finding strongly suggests a critical role for the guest molecule in facilitating these electron transfer reactions. There are two major mechanisms for charge transfer *via* molecular bridges: coherent superexchange^55^–^57^ and incoherent charge hopping.^24^ Superexchange requires the energy level(s) of the bridge engaging in this interaction to be higher than the lowest energy populated starting state of both the electron donor and acceptor, thus resulting in electron tunneling from the donor to the acceptor *via* mixing of the donor and acceptor states with the virtual bridge state. If the energy of the bridge state becomes comparable, *i.e.*, nearly resonant to that of the electron donor, a change of mechanism to thermally activated electron hopping can occur, where the electron hops to the bridge molecule for a finite time, thus destroying coherence. The total D–A coupling *V*_D,A_ in this case for bridge sites B_i_ engaged in a superexchange interaction is given by^57^2
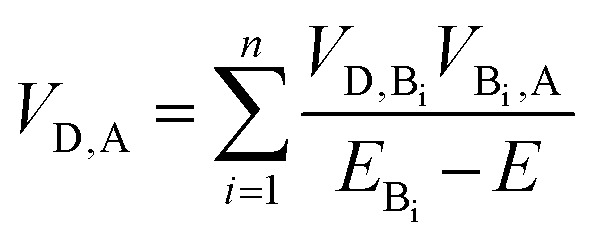
where *V*_D,B_i__ and *V*_B_i_,A_ are the coupling of the bridge state i to the donor and acceptor, respectively, *E*_B,i_ is the energy level of bridge state i, and *E* is the donor energy level. Eqn (2) reveals that the overall coupling *V*_D,A_ is inversely proportional to the energy difference between the donor and bridge states.^56^ Since the rate of electron transfer varies as the square of the coupling, the effect of superexchange diminishes as the inverse square of the energy gap. The mixing process can involve either bridge excited states or a combination of hole and/or electron transfer to the virtual states of the bridge.^57^,^58^

**Table 1:** Time constants for the indicated ICT reactions from global analysis^*a*^

Compounds	ExV^2+^–^2^*TTz^+^˙ → ExV^+^˙–TTz^2+^ (ps)	ExV^+^˙–TTz^2+^ → ExV^2+^–TTz^+^˙ (ps)
Per ⊂ TTzExVBox^4+^	N/A	16 ± 1
TTzExVBox^3+^˙	1.4 ± 0.3	135 ± 2
Per ⊂ TTzExVBox^3+^˙	0.7 ± 0.3	16 ± 1

^*a*^Experiments were performed in triplicate. Values and uncertainties are reported as the average and standard deviation, respectively. The values in the figures correspond to those for the representative data set presented.

Assuming that the FET reaction ^2^*TTz^+^˙–Per–ExV^2+^ → TTz^2+^–Per–ExV^+^˙ starts from the D_*n*_ state of ^2^*TTz^+^˙, the Per LUMO is 0.5 eV lower than the TTz^+^˙ D_*n*_ state and 1.1 eV higher than the final TTz^2+^–Per–ExV^+^˙ ion-pair state (Fig. 5b). Therefore, the factor of 2 rate enhancement observed for Per ⊂ TTzExVBox^3+^˙ relative to TTzExVBox^3+^˙ could potentially arise from electron hopping to Per, *i.e.*, ^2^*TTz^+^˙–Per–ExV^2+^ → TTz^2+^–Per^–^˙–ExV^2+^. However, if Per^–^˙ is indeed involved in the FET process, a new absorption feature should appear around 580 nm,^59^,^60^ which is not observed in Fig. 6a. On the other hand, if excitation of the TTz^+^˙ subunit leads to the formation of the ^2^*TTz^+^˙ D_1_ or Dhot0 state, which is at least 0.6 eV lower than the Per LUMO (Fig. 5b), then it is not possible for either of these ^2^*TTz^+^˙ states to reduce Per directly during the FET process. Consequently, the slightly faster FET rate in the complex is attributed to the superexchange mechanism and/or modulation of the barrier by the presence of the perylene guest.

Importantly, while the states responsible for superexchange mixing in both cases are energetically accessible from the initial photoexcited states, they are not observed to be populated during the FET process. This result is expected since both the oxidation of perylene from TTz^2+^–^1^*Per–ExV^2+^ → TTz^+^˙–Per^2+^–ExV^+^˙ and the triradical formation reaction ^2^*TTz^+^˙–Per–ExV^2+^ → TTz^+^˙–Per^+^˙–ExV^+^˙ are two-electron processes that are improbable, and hence slow.^57^,^58^

Focusing on the BET reaction, there are a multitude of states in energetic proximity to each donor to provide viable coupling through superexchange.^55^,^56^ For the TTz^2+^–Per–ExV^+^˙ → TTz^+^˙–Per–ExV^2+^ process (Fig. 5b), the perylene bridge can either be oxidized or reduced^61^ by the neighboring acceptor or donor, which yields two ionic states ∼1.36 and 1.65 eV above the donor energy level, respectively. The coupling between the donor and these two ionic states, according to eqn (2), both contribute to the total enhanced donor–acceptor coupling. The lowest excited state of the bridge is 2.85 eV above the donor state, so based on eqn (2), its contribution should be negligible.

The back-electron transfer pathway in the system TTz^2+^–Per–ExV^2+^ (Fig. 5a) is more complicated. Here, the TTz^2+^–Per^+^˙–ExV^+^˙ → TTz^+^˙–Per^+^˙–ExV^2+^ reaction competes with the direct recombination to the ground state (TTz^2+^–Per–ExV^2+^). As discussed above, this direct recombination is not observed to be the dominant pathway: if the population of TTz^2+^–Per^+^˙–ExV^+^˙ directly recombined with its own intrinsic rate, then the absorption bands associated with the TTz^+^˙ and ExV^+^˙ radicals would decay with different rates, which is not observed. Therefore, there must be some other process contributing to the observed (total) rate of decay that dominates over direct recombination. If we examine the states available for superexchange, the BET reaction may be mediated by TTz^+^˙–Per^2+^–ExV^+^˙ through hole transfer to the bridge;^57^,^58^ this state lies 0.87 eV above the donor state.^62^ Additionally, the ^2^*Per^+^˙ excited state may also assist *via* superexchange as it is 1.57 eV above the TTz^2+^–Per^+^˙–ExV^+^˙ donor.^63^ The superexchange energy levels are highlighted in Fig. 5.

The observation of the same (16 ps)^–1^ BET rate in both cases is most likely a coincidence owing to the different energies of the relevant bridge states. Since the lowest energy donor-bridge gaps in each complex are dissimilar, achieving the same BET rate would require compensation by the electronic couplings and/or by the additive nature of each pathway's contribution implied by eqn (2). It is interesting to note that the donor-bridge energy gaps between the TTz^2+^–Per^+^˙–ExV^+^˙/TTz^2+^–^2^*Per^+^˙–ExV^+^˙ (1.57 eV, Fig. 5a) and TTz^2+^–Per–ExV^+^˙/TTz^2+^–Per^–^˙–ExV^2+^ (1.65 eV, Fig. 5b) pairs are quite comparable. If the electronic coupling products *V*_D,B_i__ × *V*_B_i_,A_ are similar for both systems and are significantly larger than those for the other tunneling pathways, then according to eqn (2) the rates of BET should also be similar. However, accurately determining the relevant electronic couplings for each pathway is challenging, and so the precise origin of the rate correspondence is difficult to identify.

## Conclusions

We have presented a supramolecular complex composed of an asymmetric cyclophane TTzExVBox^4+^ that binds a Per guest, which can be used to model A–D–A′ (Per ⊂ TTzExVBox^4+^) or D–B–A (Per ⊂ TTzExVBox^3+^˙) systems for studying photoinduced electron transfer reactions. Competitive FET to both acceptors in Per ⊂ TTzExVBox^4+^ occurs when ^1^*Per serves as the electron donor. Despite the fact that the free energy change for TTz^+^˙–Per^+^˙–ExV^2+^ formation is 0.4 eV larger than that for TTz^2+^–Per^+^˙–ExV^+^˙, electron transfer to TTz^2+^ is slower compared to ExV^2+^, which is also reflected in their transient populations. The ensuing BET for TTz^2+^–Per^+^˙–ExV^+^˙ occurs faster than that in the control experiments, indicative of an indirect BET route to the TTz^2+^–Per–ExV^2+^ ground state. Studies of TTzExVBox^3+^˙ reveal that the presence of the Per guest inside the cyclophane can markedly enhance the FET and BET rates, which is ascribed to the superexchange mechanism. This research demonstrates that easily tunable supramolecular A–D–A′ or D–B–A complexes in which the donor, bridge, and acceptor components are part of a rigid, box-like cyclophane are versatile systems for studying photoinduced electron transfer in which the oxidation states of guest molecules can be precisely controlled.

## Conflicts of interest

There are no conflicts of interest to declare.

## Supplementary Material

Supplementary information

Crystal structure data
