# Supplementary material for: Choosing sides: unusual ultrafast charge transfer pathways in an asymmetric electron-accepting cyclophane that binds an electron donor
Source: Chem Sci. 2019 Mar 11;10(15):4282–92. doi: 10.1039/c8sc05514a (PMC6471873; doi:10.1039/c8sc05514a)
Supplement: Supplementary file 1 [file SC-010-C8SC05514A-s001.pdf]

Electronic Supplementary Material (ESI) for Chemical Science.
This journal is © The Royal Society of Chemistry 2019

Electronic Supplementary Information

for

Choosing Sides: Unusual Ultrafast Charge Transfer Pathways in an Asymmetric Electron-Accepting Cyclophane that Binds an Electron Donor†

Jiawang Zhou,[1,2‡] Yilei Wu,[1,2‡] Indranil Roy,[1] Avik Samanta,[1]

J. Fraser Stoddart,[1,3,4] Ryan M. Young,[1,2]* and Michael R. Wasielewski[1,2]*

[1]Department of Chemistry and [2]Institute for Sustainability and Energy,

Northwestern University, 2145 Sheridan Road, Evanston, Illinois 60208-3113

[3]Institute for Molecular Design and Synthesis, Tianjin University, Tianjin 300072, China

[4]School of Chemistry, University of New South Wales, Sydney, New South Wales 2052, Australia

†J.Z. and Y.W. contributed equally.

Table of Contents

**Section A. Materials / General Methods / Instrumentation**	**S2**
**Section B. Synthetic Protocols**	**S3**
**Section C. NMR Spectroscopies**	**S5**
**Section D. Crystallographic Characterization**	**S6**
**Section E. Steady-State and Time-Resolved Spectroscopy**	**S8**
**Section F. References**	**S18**

Section A. Materials / General Methods

All chemicals and reagents were purchased from commercial suppliers (Aldrich or Fisher) and used without further purification. 2,5-di(pyridin-4-yl)thiazolo[5,4-d]thiazole (TzBIPY), TTzExVBox•4PF_6, Bn-TTz•2PF_6 and Bn-ExV•2PF_6 were prepared according to previous literature procedures.[1, 2] Thin layer chromatography (TLC) was performed on silica gel 60 F254 (E. Merck). Column chromatography was carried out on silica gel 60F (Merck 9385, 0.040–0.063 mm). High-resolution mass spectra were measured on an Agilent 6210 Time of Flight (TOF) LC-MS, using an ESI source, coupled with Agilent 1100 HPLC stack, using direct infusion (0.6 mL/min). Nuclear magnetic resonance (NMR) spectra were recorded on a Bruker Avance 600 and Varian P-Inova 500 spectrometers, with working frequencies of 500 and 600 MHz, respectively. Chemical shifts were reported in ppm relative to the signals corresponding to the residual nondeuterated solvents (CD_3CN: δ 1.94 ppm).

UV/Vis absorption spectra were recorded using a UV-3600 Shimadzu spectrophotometer. Cyclic Voltammetry (CV) experiments were carried out at room temperature in Ar-purged solutions of dry CH_3CN with a Gamry Multipurpose instrument (Reference 600) interfaced to a PC. All CV experiments were performed using a glassy carbon working electrode (0.071 cm^2). The electrode surface was polished routinely with 0.05 µm alumina-water slurry on a felt surface immediately before use. Platinum wire (Pt) and Ag/AgCl electrode were used as counter electrode and reference electrode, respectively. The concentration of the sample and supporting electrolyte, tetrabutylammonium hexafluorophosphate (TBAPF_6), were 1.0 mM and 0.1 M, respectively. The CV cell was dried in an oven immediately before use, and Ar was continually flushed through the cell as it was cooled down to room temperature to avoid condensation of H_2O.

Section B. Synthetic Protocols

1) Bis-bromomethyl(bis-p-benzyl-4,4'-(thiazolo[5,4-d]thiazole-2,5-diyl))bis(hexafluoro-phosphate) = TTz-DB•2PF$_6$

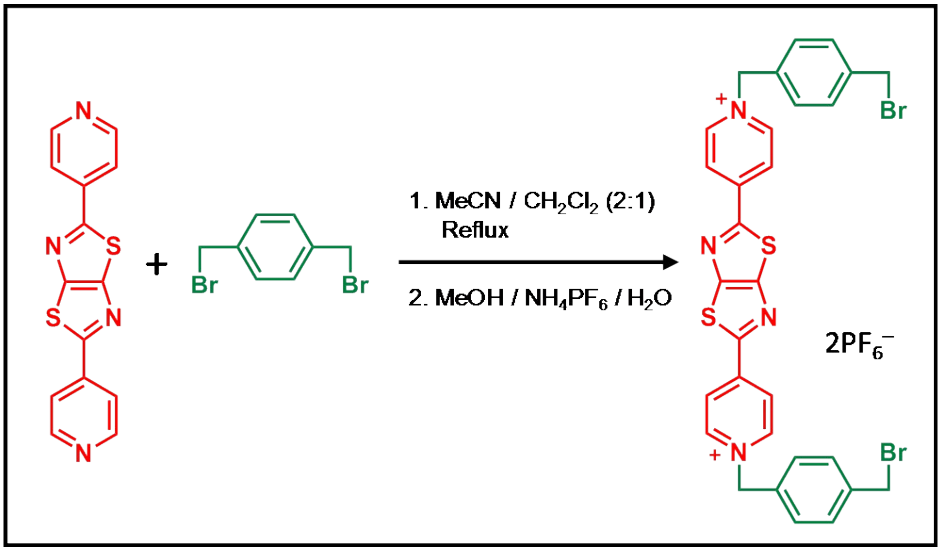

Scheme S1. Synthesis of TTz-DB•2PF$_6$ (5•2PF$_6$)

The synthetic protocol was adapted from the literature procedure.[1] *α,α*-Dibromo-*p*-xylene (3.69 g, 13.86 mmol) was introduced into a round-bottomed flask and dry CH_2Cl_2 (30 mL) was added to the flask. The mixture was heated under reflux at 50 °C to obtain a clear solution. Once all the compounds had dissolved the temperature was raised to 90 °C. TzBIPY (409 mg, 1.38 mmol) was dissolved in dry CH_3CN (60 mL) and added to the reaction mixture over 1 h (4-5 portions). The yellow precipitate started forming after 30 min. The reaction mixture was stirred for 2 days at 90 °C, then it was brought to the room temperature and the yellow precipitate was filtered off and washed with CH_2Cl_2 to remove the unreacted starting materials. Finally, the solid was dissolved in H_2O and an excess of NH_4PF_6 was added to precipitate the crude product. Excess NH_4PF_6 was removed by washing several times with H_2O to obtain the pure whitish yellow product in ca. 80% yield. ^{1}H NMR (500 MHz, CD_3CN, 25 ºC): δ = 8.84 (AA′ of AA′XX′, J = 6.5

Hz, 4H), 8.54 (XX′ of AA′XX′, J = 6.5 Hz, 4H), 7.55 (AA′ of AA′BB′, J = 7.8 Hz, 4H), 7.47 (BB′ of AA′BB′, J = 7.8 Hz, 4H), 5.75 (s, 4H), 4.61 (s, 4H). ^{13}C NMR (125 MHz, CD_3CN, 25 ºC): δ = 166.0, 158.5, 157.1, 146.6, 141.2 133.8, 131.2, 130.6, 125.8, 64.9, 33.5.

2) TTzBox•4PF_6

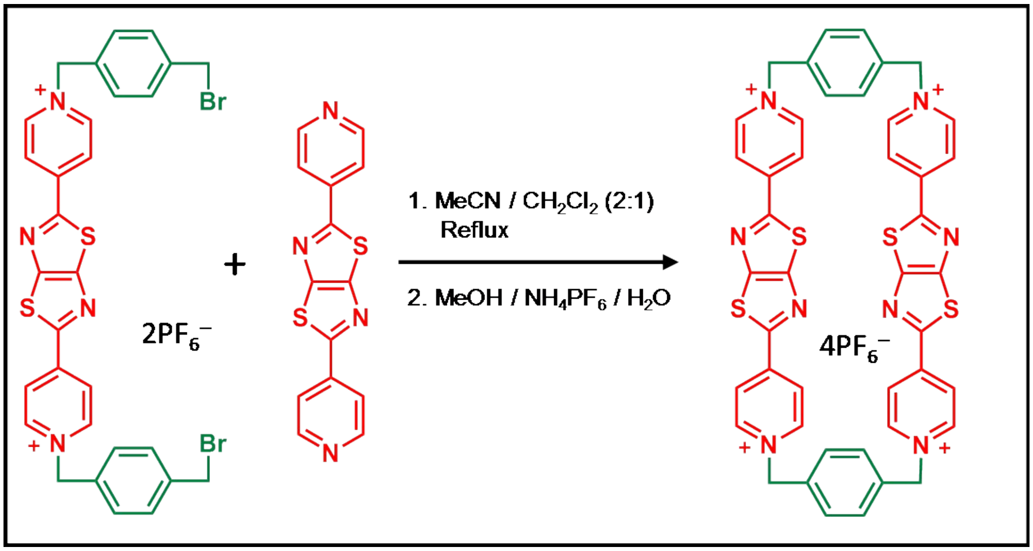

Scheme S2. Synthesis of 6•4PF_6.

TTz-DB•2PF_6 (0.191 g, 0.20 mmol), TzBIPY (59 mg, 0.20 mmol) and TBAI (0.016 g) were introduced into round-bottomed flask and dry CH_3CN (180 mL) was added to the flask. The mixture was stirred at 80 °C for 4 days. The reaction mixture was brought to room temperature and excess of NH_4Cl was added to precipitate a yellow solid. The precipitate was filtered off and washed with Me_2CO and CH_2Cl_2 to remove the tetrabutylammonium salt. The solid was dried and then dissolved in H_2O, and reprecipitated as its PF_6^- salt by adding solid NH_4PF_6 (~5% (w/v)). The excess NH_4PF_6 was removed by multiple washes with H_2O. Finally, pure TTzBox•4PF_6 was obtained after running a reverse-phase chromatography column using H_2O/ CH_3CN (9:1 v/v) in 25% yield as a yellow solid. ^{1}H NMR (500 MHz, CD_3CN, 25 ºC) δ = 8.86

(d, 6.8 Hz, 8H), 8.36 (d, 6.8 Hz, 8H), 7.62 (d, 8 Hz, 8H), 5.72 (s, 8H). ^{13}C NMR (125 MHz, $CDCl_3$, 25 ºC) δ = 165.7, 156.9, 148.2, 146.0, 137.0, 131.1, 125.8, 65.0.

Section C. NMR Spectroscopies

^{1}H NMR Spectra in CD_3CN:

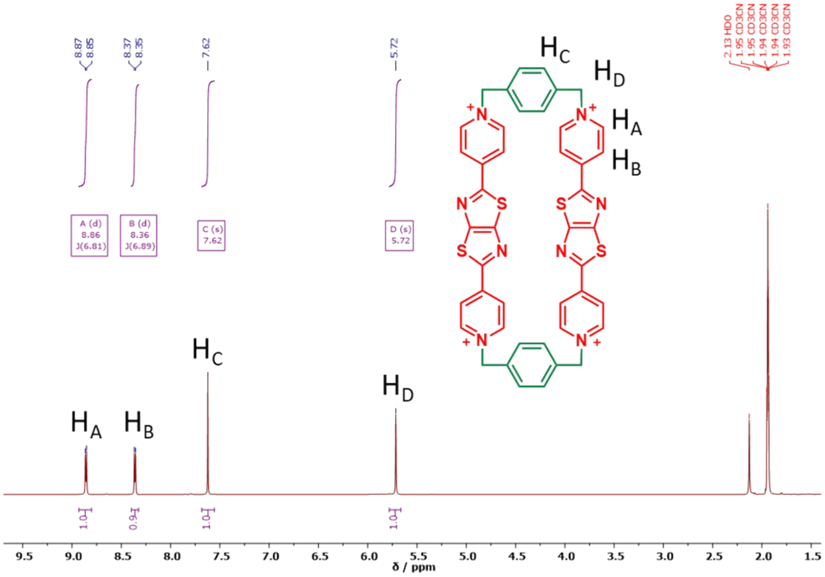

Figure S1. Annotated ^{1}H NMR spectrum (500 MHz, CD_3CN, 25 ºC) of TTxBox•4PF_6.

^{13}C NMR Spectra in CD_3CN:

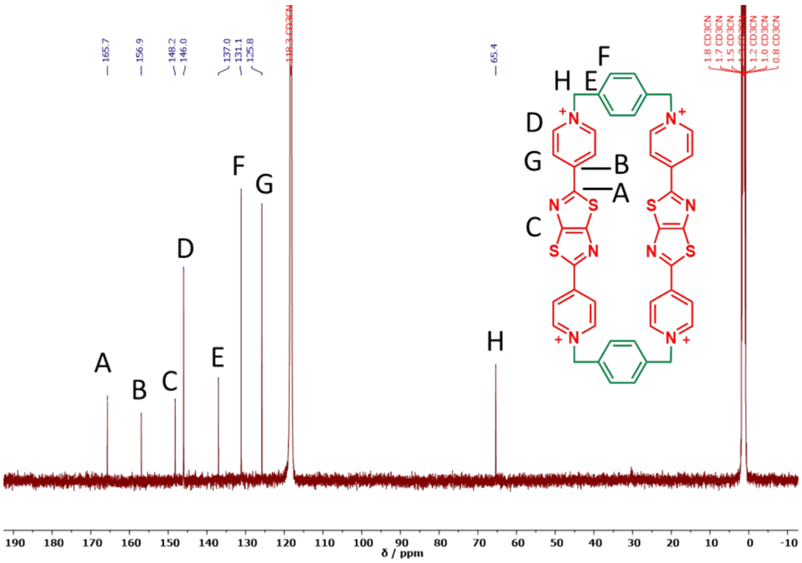

Figure S2. Annotated ^{13}C NMR spectrum (500 MHz, CD_3CN, 25 ºC) of TTzBox•4PF_6.

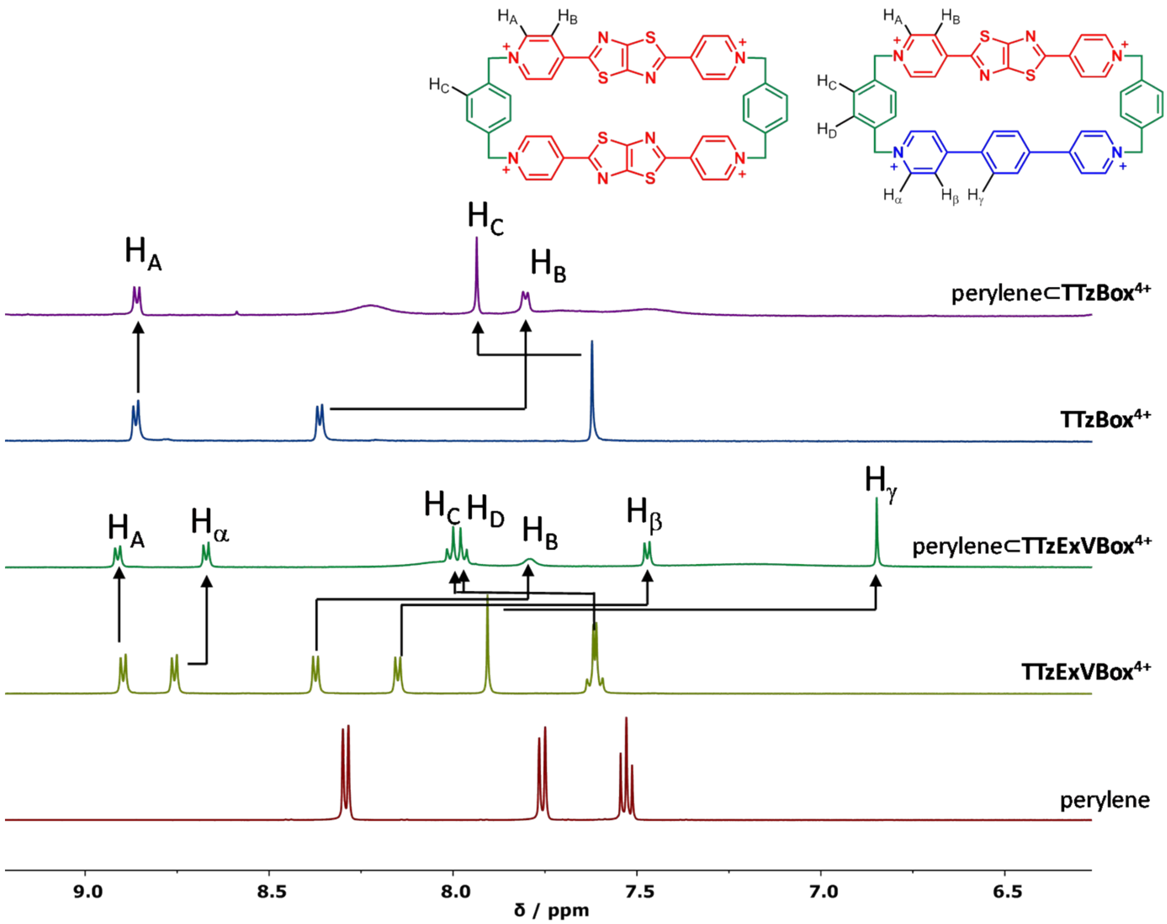

Figure S3. Aromatic-region insets of the ^{1}H NMR spectra of hosts and the Per guest and its 1:1 complexes recorded in CD_3CN at 298 K on a 500 MHz instrument. Upfield shifts of the aromatic protons of ExV^{2+} and TTz^{2+} subunits as well as downfield shifts of the *p*-xylene protons are evident, indicating the binding of a Per inside $TTzExV^{4+}$ and $TTzBox^{4+}$ in solution.

Section D. Crystallographic Characterization

A suitable crystal was selected and the crystal was mounted on a MITIGEN holder in Paratone oil on a Kappa Apex 2 diffractometer. The crystal was kept at 99.93 K during data collection. Using Olex2,[3] the structure was solved with the ShelXT[4] structure solution program using Direct Methods and refined with the ShelXL[5] refinement package using Least Squares minimization.

The crystallographic data for Per⊂TTzBox•4PF_6 (CCDC 1872160) and for Per⊂TTzExVBox•4PF_6 (CCDC 1872161) are available free of charge from the Cambridge Crystallographic Data Centre via www.ccdc.cam.ac.uk/data_request/cif.

1) Per⊂TTzExVBox•4PF_6

a) Method: Single crystals of Per⊂TTzExVBox•4PF_6 were grown by slow vapor diffusion of $^{i}Pr_2O$ into a solution of Per⊂TTzExVBox•4PF_6 in CH_3CN over the course of 3 days. A suitable single crystal was selected and mounted in inert oil and transferred to the cold gas stream of a Kappa Apex 2 diffractometer. The crystal was kept at 100 K during data collection. Using Olex2, the structure was solved with the ShelXT structure solution program using Direct Methods and refined with the ShelXL refinement package using Least Squares minimization.

b) Crystal Data: for $C_{90}H_{66}F_{24}N_8P_4S_2$ (M =1903.50): triclinic, space group P-1 (no. 2), a = 10.448(2) Å, b = 10.636(2) Å, c = 19.123(4) Å, α = 101.538(10)°, β = 95.067(11)°, γ = 104.449(10)°, V = 1994.8(7) Å^3, Z = 1, T = 100.0 K, μ(CuKα) = 2.353 mm^{-1}, $Dcalc$ = 1.585 g/mm^3, 16091 reflections measured ($4.768 \leq 2\Theta \leq 130.29$), 6669 unique ($R_{int}$ = 0.0675, R_{sigma} = 0.0599) which were used in all calculations. The final R_1 was 0.0760 ($I > 2\sigma(I)$) and wR_2 was 0.1943 (all data).

c) Refinement Details: Distance restraints were imposed on the disordered Nitrogen-Carbon and Sulfur-Carbon distances. The enhanced rigid-bond restraint (SHELX keyword RIGU) was applied globally.[6] There is a full molecule disorder on the Box.

2) Per⊂TTzBox•4PF_6

a) Method: Single crystals of Per⊂TTzBox•4PF_6 were grown by slow vapor diffusion of $^{i}Pr_2O$ into a solution of Per⊂TTzBox•4PF_6 in CH_3CN over the course of 3 days. A suitable single crystal was selected and mounted in inert oil and transferred to the cold gas stream of a Kappa

Apex 2 diffractometer. The crystal was kept at 100 K during data collection. Using Olex2, the structure was solved with the ShelXT structure solution program using Direct Methods and refined with the ShelXL refinement package using Least Squares minimization.

b) Crystal Data for $C_{88}H_{62}F_{24}N_{10}P_4S_4$ (M =1967.59): triclinic, space group P-1 (no. 2), a = 10.3592(4) Å, b = 10.5764(4) Å, c = 19.6578(8) Å, α = 102.138(2)°, β = 95.368(2)°, γ = 103.900(2)°, V = 2020.28(14) Å^3, Z = 1, T = 100.01 K, μ(CuKα) = 2.823 mm^{-1}, $Dcalc$ = 1.617 g/mm^3, 33529 reflections measured ($8.874 \leq 2\Theta \leq 130.354$), 6894 unique ($R_{int}$ = 0.0335, R_{sigma} = 0.0256) which were used in all calculations. The final R_1 was 0.0370 (I > 2σ(I)) and wR_2 was 0.1019 (all data).

c) Refinement Details: The enhanced rigid-bond restraint (SHELX keyword RIGU) was applied on the disordered atoms.

Section E. Steady-State and Time-Resolved Spectroscopy

All transient absorption data were background-subtracted to remove scattered light and thermally lensed fluorescence from the spectra, and then corrected for group delay dispersion (GDD, or "chirp") using Surface Xplorer Pro 4 (Ultrafast Systems, LLC). Kinetic traces were fit (singly or globally) in a laboratory-written MATLAB program.[7] The program solves the differential equations of the specified kinetic model, then convolutes them with a Gaussian instrument response function before employing a least-squares fitting to iteratively find the parameters which result in matches to the same functions for all selected wavelengths. Once these parameters are established, they are fed into the model solutions to yield the populations of each state in model. Finally, the total raw data matrix is deconvoluted with these populations as a function of time to produce the spectra associated with each species.

We used the following first-order kinetic models with rate matrices K for the compounds and excitation wavelengths:

For the fsTA of Per⊂TTzExVBox^{4+} and Per⊂TTzBox^{4+} exciting at 414 and 450 nm:

$$\underline{\underline{K}} = \begin{pmatrix} -k_A & 0 & 0 \\ 0 & -k_B & 0 \\ 0 & 0 & -k_C \end{pmatrix} \qquad \text{(Eqn. S1)}$$

where k_A, k_B and k_C represent the rates of formation of the CS state, the decay of the CS state and of the perylene S_1 state, respectively. A species-associated model was initially employed to fit the data, but it was not able to successfully separate the S_1 spectra between the free perylene and the perylene encapsulated within the cyclophane. Therefore, a decay-associated model was used instead.

For the fsTA of Bn-TTz$^{+\bullet}$ exciting at 620 nm:

$$\underline{\underline{K}} = \begin{pmatrix} -k_A & 0 \\ k_A & -k_B \end{pmatrix} \qquad \text{(Eqn. S2)}$$

where A and B represent the D_n and D_1 states, respectively.

For the fsTA of Per⊂TTzExVBox$^{3+\bullet}$ exciting at 620 nm:

$$\underline{\underline{K}} = \begin{pmatrix} -k_A & 0 \\ k_A & -k_B \end{pmatrix} \qquad \text{(Eqn. S3)}$$

where A and B represent the D_n and CS states, respectively.

For the fsTA of TTzExVBox$^{3+\bullet}$ exciting at 620 nm:

$$\underline{\underline{K}} = \begin{pmatrix} -k_A - k_B & 0 & 0 \\ -k_A & -k_C & 0 \\ -k_B & 0 & -k_D \end{pmatrix} \qquad \text{(Eqn. S4)}$$

where k_A and k_B represent the rates for formation of the D_1 and CS states from D_n, respectively. For simplicity, k_A is fixed to $(0.8\ ps)^{-1}$ based on the value from the fsTA of Bn-TTz$^{+\bullet}$. C and D represent the D_1 and CS states, respectively.

c) Transient Absorption Spectroscopy:

The time-resolved differential absorption spectra are reported in Figures 6, 8-10 in the main text and S8, S12-S14, while the kinetic analyses are presented in Figures S7-S14.

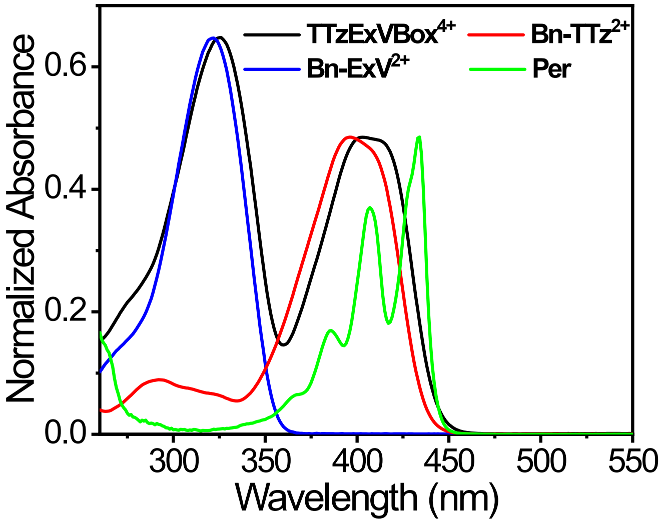

Figure S4. Steady-state absorption spectra of TTzExVBox^{4+}, Bn-TTz^{2+}, Bn-ExV^{2+}and Per in CH_3CN.

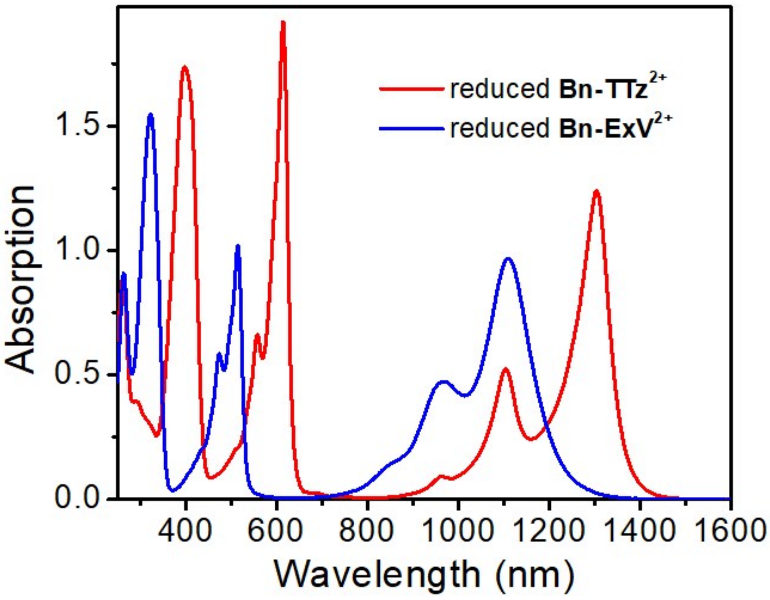

Figure S5. Steady-state absorption spectra of excess Bn-ExV^{2+} and Bn-TTz^{2+} chemically reduced by equal amount of $CoCp_2$ in CH_3CN. The absorption bands at 330 and 405 nm signify the residual Bn-ExV^{2+} and Bn-TTz^{2+}, respectively.

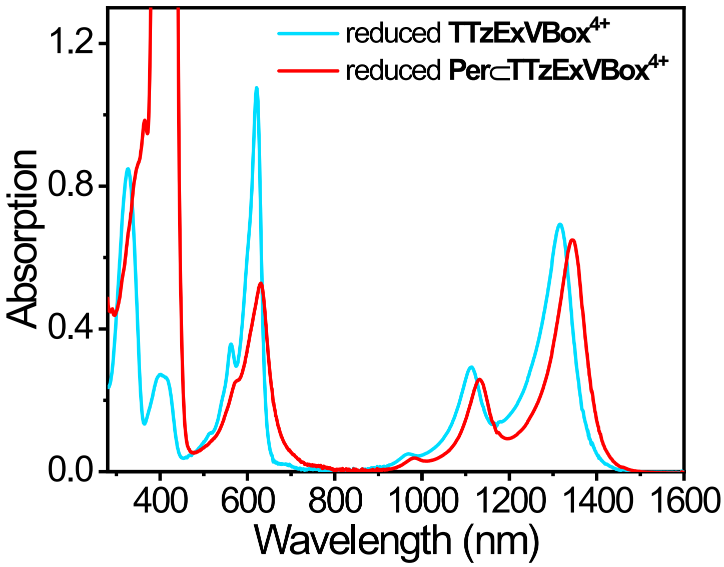

Figure S6. Steady-state absorption spectra of TTzExVBox^{4+} and Per⊂TTzExVBox^{4+} chemically reduced by $CoCp_2$ in CH_3CN. The amount of $CoCp_2$ was less than one molar equivalent of either TTzExVBox^{4+} or Per⊂TTzExVBox^{4+}.

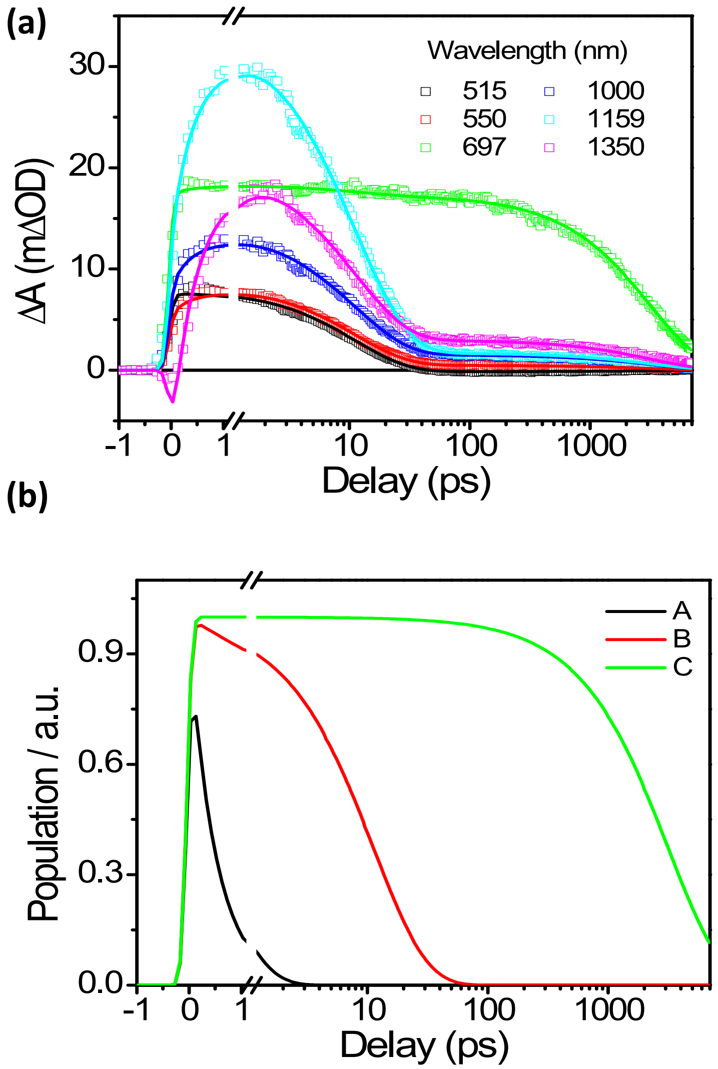

Figure S7. (a) Multiple-wavelength fits and (b) populations of kinetic states of Per⊂TTzExVBox^{4+} in CH_3CN excited at 414 nm. (A: formation of CS state, B: decay of CS state, C: Per S_1)

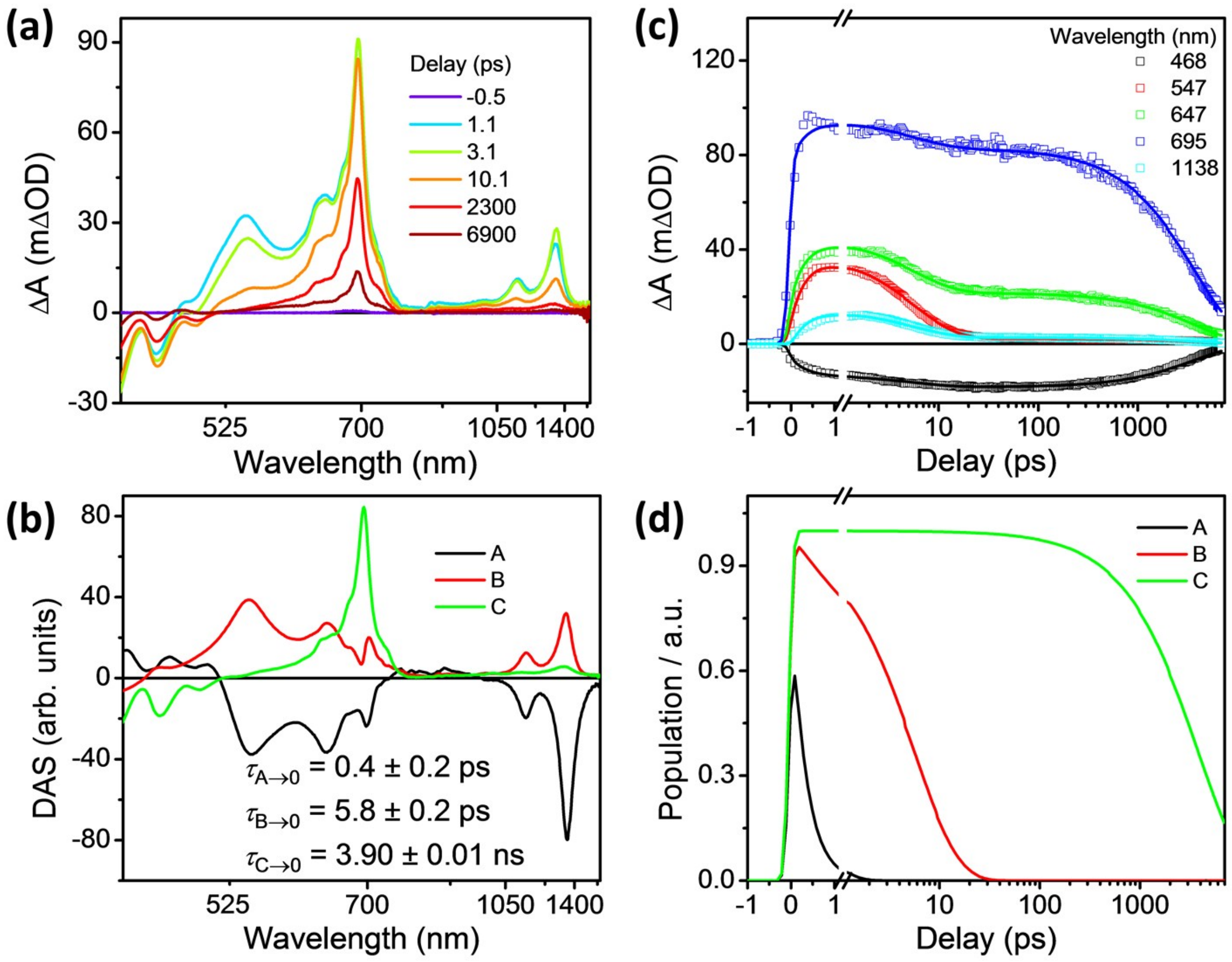

Figure S8. (a) fsTA spectra and (b) decay-associated spectra (DAS) and (c) Multiple-wavelength fits and (d) populations of kinetic states of Per⊂TTzBox^{4+} in CH_3CN excited at 414 nm. (A: formation of CS state, B: decay of CS state, C: Per S_1)

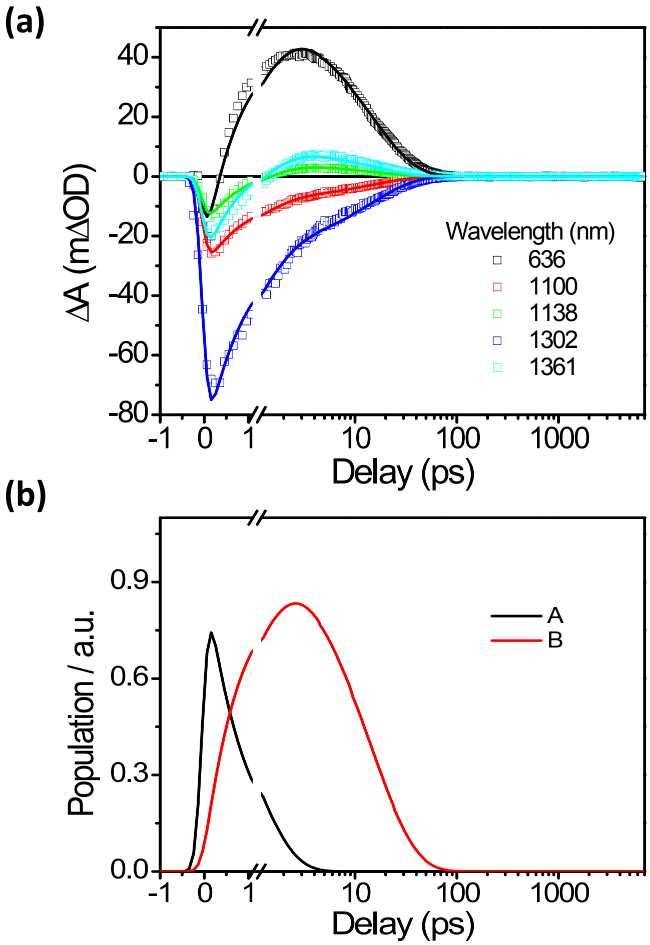

Figure S9. (a) Multiple-wavelength fits and (b) populations of kinetic states of Bn-TTz$^{+•}$ in CH_3CN excited at 620 nm. (A: D_n, B: D_1)

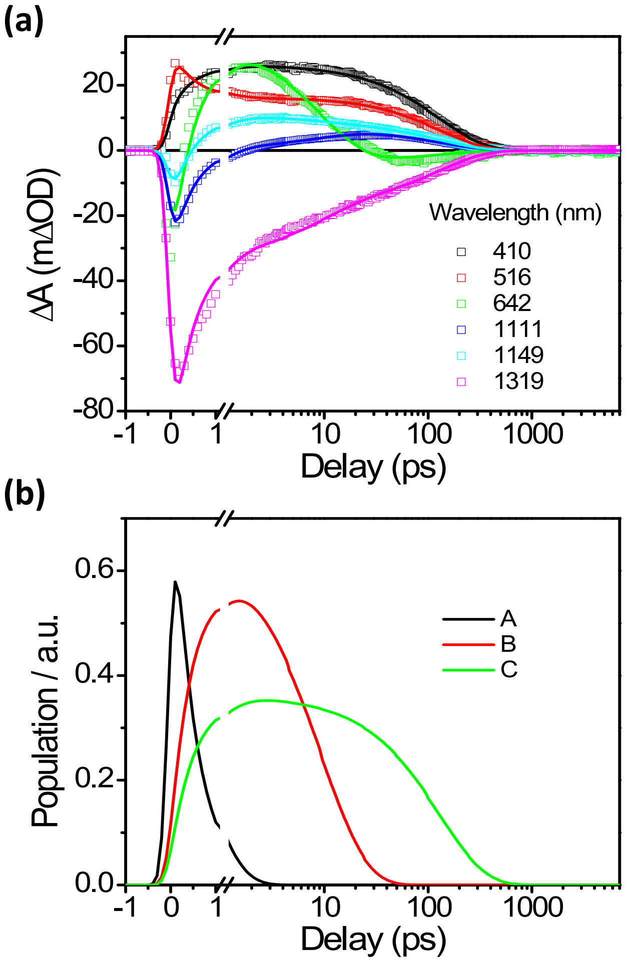

Figure S10. (a) Multiple-wavelength fits and (b) populations of kinetic states of TTzExVBox$^{3+\bullet}$ in CH_3CN excited at 620 nm. (A: D_n, B: D_1, C: CS state)

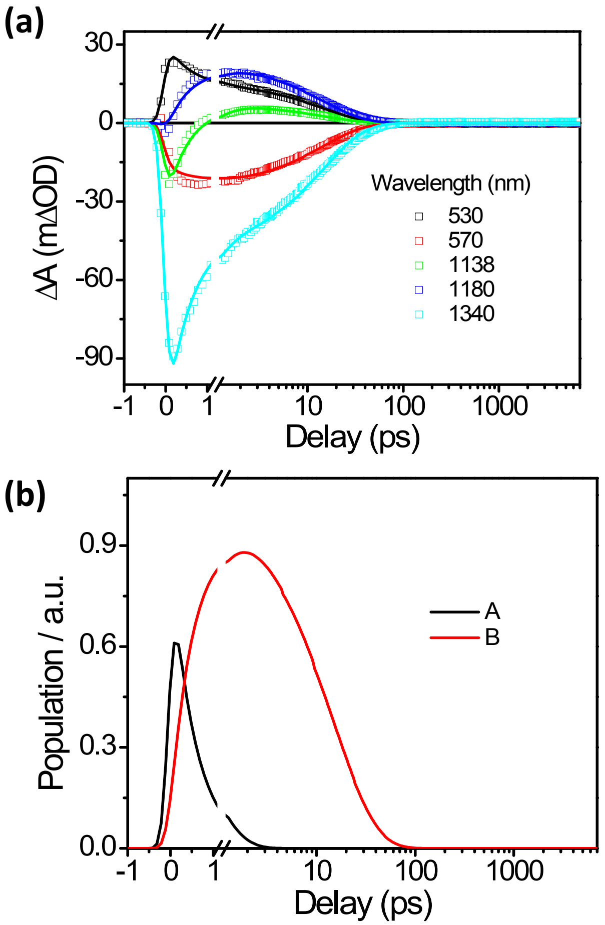

Figure S11. (a) Multiple-wavelength fits and (b) populations of kinetic states of Per⊂TTzExVBox$^{3+•}$ in CH_3CN excited at 620 nm. (A: D_n, B: CS state)

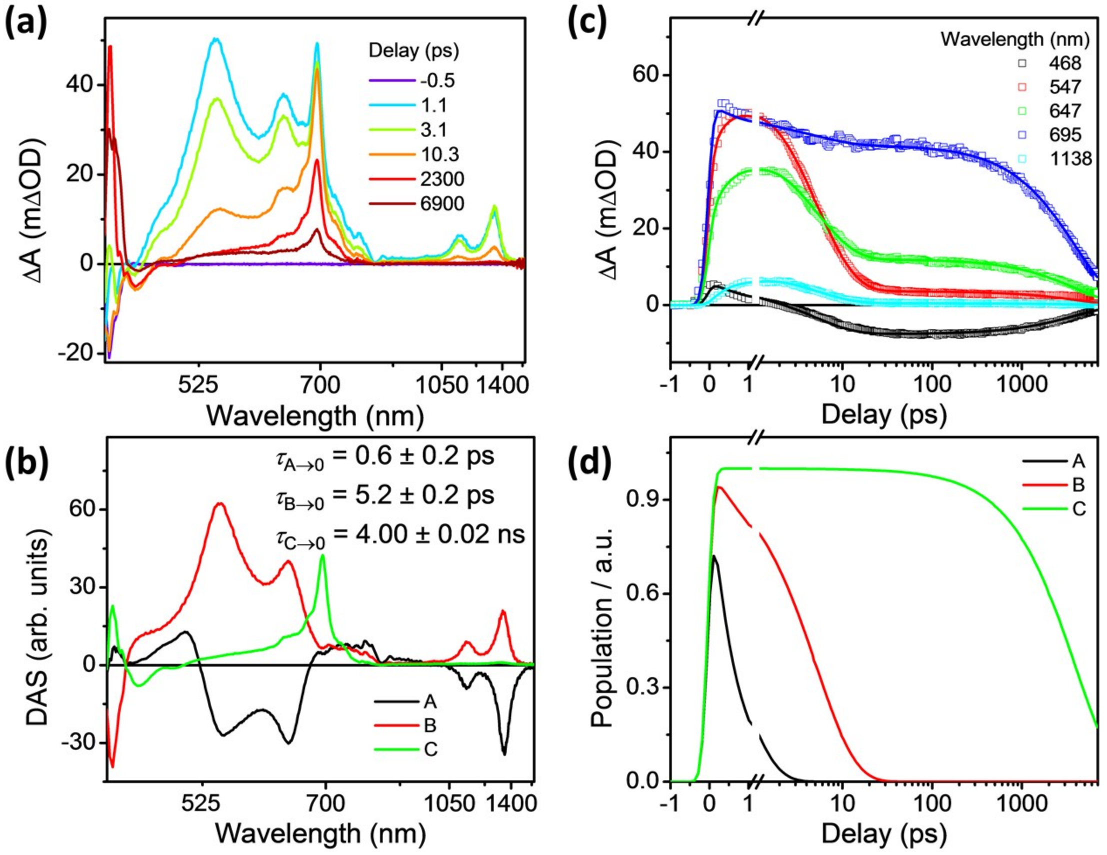

Figure S13. (a) fsTA spectra and (b) decay-associated spectra (DAS) and (c) Multiple-wavelength fits and (d) populations of kinetic states of Per⊂TTzBox^{4+} in CH_3CN excited at 450 nm. (A: formation of CS state, B: decay of CS state, C: Per S_1)

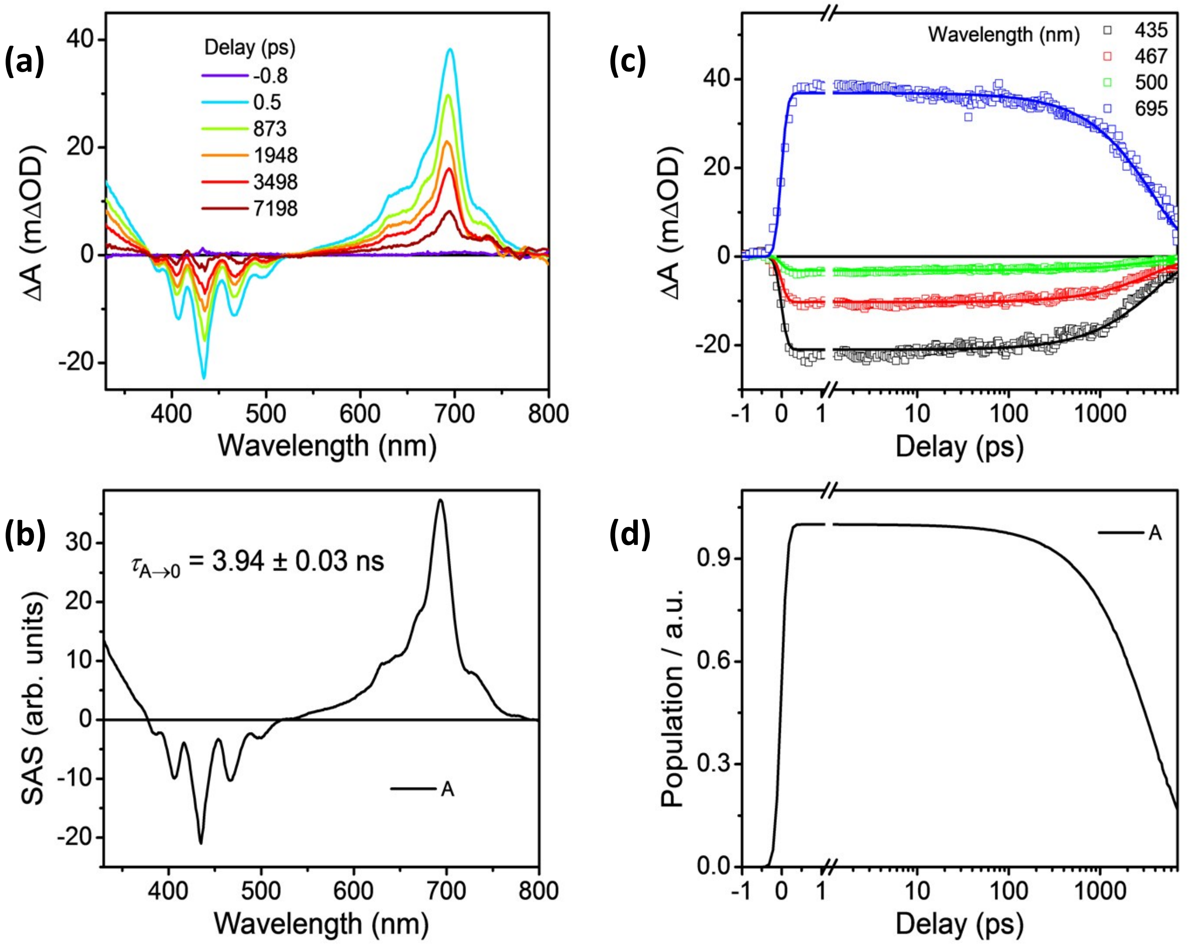

Figure S14. (a) fsTA spectra and (b) species-associated spectra (SAS) and (c) Multiple-wavelength fits and (d) populations of kinetic states of Per in CH_3CN excited at 414 nm. (A: Per S_1)

Section F. References

1. J. C. Barnes, M. Juricek, N. L. Strutt, M. Frasconi, S. Sampath, M. A. Giesener, P. L. McGrier, C. J. Bruns, C. L. Stern, A. A. Sarjeant and J. F. Stoddart, *J. Am. Chem. Soc*, 2013, **135,** 183-192.
2. I. Roy, S. Bobbala, J. Zhou, M. T. Nguyen, S. K. M. Nalluri, Y. Wu, D. P. Ferris, E. A. Scott, M. R. Wasielewski and J. F. Stoddart, *J. Am. Chem. Soc*, 2018, **140,** 7206-7212.
3. O. V. Dolomanov, L. J. Bourhis, R. J. Gildea, J. A. K. Howard and H. Puschmann, *J. Appl. Crystallogr*, 2009, **42,** 339-341.
4. G. M. Sheldrick, *Acta Crystallogr. C*, 2015, **71,** 3-8.
5. G. M. Sheldrick, *Acta Crystallogr. C*, 2015, **71,** 3-8.
6. A. Thorn, B. Dittrich and G. M. Sheldrick, *Acta Crystallogr. A*, 2012, **68,** 448-451.
7. MATLAB, 2018, The Mathworks, Inc., Natick, Massachusetts, United States.
